# Selective Deletion of NBCe1 in Reactive Astrocytes Attenuates Ischemic Stroke Brain Damage

**DOI:** 10.1002/glia.70075

**Published:** 2025-08-05

**Authors:** Okan Capuk, Elise Berthold, Kathiravan Kaliyappan, Mansi Avunoori, Rajesh Muduganti, Sanjana Krishna, Shamseldin Metwally, Mary McFarland, Shanshan Song, Victoria Fiesler, Sydney Fischer, Lesley M. Foley, T. Kevin Hitchens, Susannah Waxman, Ian A. Sigal, Shefeeq M. Theparambil, Gulnaz Begum

**Affiliations:** ^1^ Department of Neurology, the Pittsburgh Institute for Neurodegenerative Diseases University of Pittsburgh Pittsburgh Pennsylvania USA; ^2^ Carnegie Mellon University Department of Biological Sciences Pittsburgh Pennsylvania USA; ^3^ Veteran's Affairs Research Center Pittsburgh Pennsylvania USA; ^4^ Advanced Imaging Center University of Pittsburgh Pittsburgh Pennsylvania USA; ^5^ Department of Neurobiology University of Pittsburgh Pittsburgh Pennsylvania USA; ^6^ Department of Ophthalmology University of Pittsburgh Pittsburgh Pennsylvania USA; ^7^ Department of Biomedical and Life Sciences Lancaster University Lancaster UK

**Keywords:** AQP4, astrocytic end‐feet, brain pH homeostasis, ischemic stroke, NBCe1

## Abstract

The electrogenic sodium bicarbonate transporter 1 (NBCe1/*Slc4a4*), predominantly expressed in astrocytes, is important for brain pH regulation and homeostasis. Increased NBCe1 expression in reactive astrocytes has been associated with neuronal degeneration in ischemic stroke. However, the effects of astrocytic NBCe1 inhibition in stroke remain contradictory, and the underlying mechanisms are unclear. Here, we show that wild‐type (WT) mice exhibited elevated NBCe1 expression in the peri‐lesional regions at 3 days post‐stroke. Astrocytic *Nbce1* gene deletion in inducible *Gfap‐Cre* ^ERT2+/−^; *Nbce1* ^f/f^ mice (*Nbce1* ^iΔAstro^) resulted in a significant reduction in NBCe1 mRNA and protein expression in astrocytes. Compared to WT stroke mice, *Nbce1* ^iΔAstro^ mice displayed reduced infarct volume, decreased brain swelling, improved cerebral blood flow, and accelerated neurological function recovery in the 1–5‐day acute post‐stroke period. Moreover, *Nbce1* ^iΔAstro^ stroke mice exhibited decreased blood–brain barrier (BBB) permeability, accompanied by preserved perivascular AQP4 polarization, upregulation of Kir4.1 protein expression, and reduced astrocyte domain volume. Importantly, *Nbce1* ^iΔAstro^ stroke brains revealed an anti‐inflammatory cytokine profiling signature, marked by increased TIMP‐1 expression. Together, our findings suggest that astrocytic upregulation of pH regulatory protein NBCe1 after stroke contributes to increased BBB permeability, reactive astrogliosis, inflammation, and perivascular AQP4 dysregulation. Targeting astrocytic NBCe1 may represent a promising new therapeutic strategy to mitigate astroglial dysfunction in the post‐stroke brain.

## Main Points

- NBCe1 is upregulated in reactive astrocytes following ischemic stroke. Deletion of astrocytic Nboe1 reduces stroke volume, preserves AQP4 polarization, reduces BBB permeability, and improves neurological function after ischemic stroke.

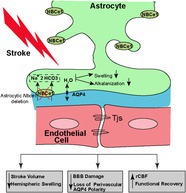

## 1 Introduction

The blood brain barrier (BBB) is essential for regulating ion and water homeostasis, nutrient transport, and cerebral hemodynamics, which is crucial for maintaining optimal brain function (Liebner et al. 2018; Profaci et al. 2020; Sweeney et al. 2019). BBB disruption is a prominent feature of acute ischemic stroke injury and is strongly associated with poor neurological recovery, hemorrhagic complications, and a high rate of mortality (Bani‐Sadr et al. 2023; Okada et al. 2020). Recent longitudinal cohort studies suggest that BBB breakdown is an early biomarker of cognitive dysfunction in humans and a key mechanism underlying age‐related cognitive decline (Barisano et al. 2022; Nation et al. 2019). Both pre‐clinical and clinical studies have reported that BBB permeability can remain elevated for weeks to several months after ischemic stroke, posing a significant risk for cognitive impairment and dementia (Desilles et al. 2013; Kassner and Merali 2015; Lee et al. 2017). BBB disruption increases neuroinflammation and damages the barrier's structural components, including tight junctions, pericytes, astrocytes, endothelial cells, and the basement membrane (Okada et al. 2020). However, the precise molecular mechanisms modulating BBB damage after ischemic stroke remain poorly understood.

The astrocyte endfeet that cover the microvessel walls of the BBB express various ion channels and transporters, receptors, and enzymes that are critical for ion and osmotic homeostasis and BBB regulation (Diaz‐Castro et al. 2023; Stokum et al. 2021). Ischemic stroke causes these endfeet to swell, resulting in a reduction of coverage of the microvessel walls (Williamson et al. 2021). These changes contribute to increased BBB permeability and cell death in the peri‐infarct area during the sub‐acute phase of stroke (Williamson et al. 2021). Previous studies also show that disruption of astrocyte ion homeostasis, characterized by elevated Ca^2+^ signaling and dysregulation in Na^+^, K^+^, H^+^, Cl^−^, and glutamate levels, occurs early after a stroke and is known to cause BBB damage (Begum et al. 2018; Stokum et al. 2015). We recently reported that astrocyte selective deletion of *Nhe1* in *Gfap‐Cre* ^ERT2+/−^; *Nhe1* ^f/f^ mice reduced ischemic infarction and edema, abolished stroke‐mediated astrogliosis, reduced expression of pro‐inflammatory protease MMP‐9, and preserved BBB function (Begum et al. 2018). We have shown that astrocytic *Nhe1* deleted mice have decreased transcellular and paracellular BBB leakage along with increased angiogenesis and cerebral perfusion after ischemic stroke. These effects result from Wnt/*β*‐catenin signaling activation in the cerebral vessels from astrocyte‐derived Wnt7a (Song et al. 2021). Our findings suggest that modulating astrocytic *Nhe1* gene activity could shift dysfunctional astrocytes toward a protective role for cerebral vascular repair after ischemic stroke. Preserving astrocyte function by preventing the overactivation of ion transporters has emerged as a potential strategy to maintain BBB homeostasis, facilitate vascular repair, and enhance neuroprotection following ischemic stroke. In a mouse model of ischemic stroke, excessive activation of SUR1‐TRPM4 and NCX1 in astrocyte endfeet led to Na^+^ and Ca^2+^ influx and altered polarization of aquaporin 4 (AQP4), resulting in BBB damage and brain swelling (Stokum et al. 2023), indicating the important role of astrocytes in maintaining BBB ion and water homeostasis.

Another astrocytic pH regulator is electrogenic sodium bicarbonate transporter 1 (NBCe1), a major HCO_3_^−^ transporter that is expressed predominantly in astrocytes and plays an important role in pH buffering in the brain (Khakipoor et al. 2019; Theparambil et al. 2020; Theparambil et al. 2014). Membrane depolarization and elevated extracellular HCO_3_^−^ causes an inward mode activation of NBCe1 (influx of HCO_3_^−^ and Na^+^), which can dampen the intracellular acidification and cause Na^+^‐mediated astrocyte swelling (Larsen and MacAulay 2017; Theparambil et al. 2015). Increased glycolytic stimulation and inhibition of oxidative phosphorylation (OxPhos) can stimulate lactate formation, which is also associated with NBCe1‐mediated HCO_3_^−^ influx (Fernandez‐Moncada et al. 2018; Ruminot et al. 2011; Theparambil et al. 2016). Despite our knowledge of these important identified physiological functions, how the activity of NBCe1 is regulated under pathological conditions in reactive astrocytes and its impact on brain functions remains largely unknown.

In this study, to investigate the astrocyte specific function of NBCe1 in ischemia‐induced brain damage, we established inducible astrocytic *Nbce1* conditional knockout mice (*Nbce1* ^iΔAstro^). We found that NBCe1 expression is significantly upregulated in reactive astrocytes in WT brains at 3 days after ischemic stroke, accompanied by loss of astrocytic endfeet AQP4 expression, BBB damage, hemispheric swelling, and large infarction. Selective deletion of *Nbce1* in astrocytes preserved polarized AQP4 expression at the astrocytic endfeet, BBB integrity, and improved neurological function recovery. Deletion of astrocytic *Nbce1* mitigates astrocyte dysfunction and promotes an anti‐inflammatory profile, suggesting that NBCe1 deficiency confers protective effects via preserving astrocyte function under ischemic conditions. Collectively, our findings establish the important role of astrocytic NBCe1 in the functional regulation of the BBB following ischemic injury.

## 2 Materials and Methods

### 2.1 Materials

Tamoxifen (#T5648), corn oil (#C8267), Evans blue dye (#E2129), DiI (42364) and DAPI (4′, 6‐Diamidino‐2‐Phenylindole Dihydrochloride) (#D1306) and low gelling temperature agarose (#A9045) were from Sigma‐Aldrich (St. Louis, MO). Adult Brain Dissociation Kit, mouse and rat (#130‐107‐677) and Anti‐ACSA‐2 MicroBead Kit, mouse (#130‐097‐679) were from Miltenyi Biotec (Germany). RNAscope Multiplex Fluorescence Assay kit, Slc4a4 probe (#533358), mouse 3‐plex positive probe (#320881), 3‐plex negative probe (#320871) and proLong Gold Antifade Mountant (#ZG0729) were from Advanced Cell Diagnostics (Hayward, CA). Pierce BCA Protein Assay kit (#23227), West Pico PLUS Chemiluminescent Substrate (#34580) and pHrodo Red AM (Cat. #P35372) were from ThermoFischer Scientific (Waltham, MA). DiO (#D275) and DiD (#D7757) were from Invitrogen (Carlsbad, CA). Mouse Cytokine Array Kit (#ARY006) was from R&D systems Inc. (Minneapolis, MN). Gold microparticles (#1652263), Tefzel tubing (#1652441), tubing preparation station (#1652418) and Helios Gene Gun (#1652411) were purchased from Bio‐Rad (Hercules, CA). All antibodies used in this study are listed in the Table S1.

### 2.2 Animals

Floxed *Nbce1* mice were obtained from the University of Cincinnati (Vairamani et al. 2018) and genotyped for the presence of the floxed or wild‐type *Slc4a4* allele using a PCR‐based method as described previously (Brown et al. 2021). Primers designed for the flanking proximal *LoxP* site (F; 5′‐TGGTGGCTTAAATTGCAAATGGC‐3′; R: 5′‐CATAACCCACTAAGTCCAGTACG‐3′) yield a 223 bp product for the floxed allele and 176 bp for the wild‐type allele (Vairamani et al. 2018). The inducible *Gfap‐Cre* ^ERT2+/−^ (*B6.Cg‐Tg(GFAP‐cre/ERT2)505Fmv/J*; Jackson Laboratory# 012849) mice with the expression of a Cre‐estrogen receptor (Cre‐ERT2) fusion to a human glial fibrillary acidic protein (GFAP) promoter sequence in mature and immature astrocytes (Ganat et al. 2006) were genotyped as described previously (Begum et al. 2018). *Nbce1* ^f/f^ mice were cross bred with *Gfap‐Cre* ^ERT2+/−^ mice to generate *Gfap‐Cre* ^ERT2+/−^; *Nbce1* ^f/f^ mice. Astrocyte specific inducible *Nbce1* knockout mice (*Nbce1* ^iΔAstro^) were generated by a 5 day injection of tamoxifen (Tam) in corn oil at a dosage of 75 mg/kg (i.p.) starting at postnatal day 60 to 90 (P60‐90). *Gfap‐Cre* ^ERT2−/−^; *Nbce1* ^f/f^ mice injected with Tam served as controls.

Animals were provided with food and water ad libitum and maintained in a temperature‐controlled environment with a 12/12 h light–dark cycle. All studies were in compliance with the guidelines outlined in the Guide for the Care and Use of Laboratory Animals from the U.S. Department of Health and Human Services and were approved by the University of Pittsburgh Institutional Animal Care and Use Committee. Surgeries and all outcome assessments were performed by investigators blinded to mouse genotype and experimental group assignments.

### 2.3 Focal Ischemic Stroke Model

Transient focal ischemic stroke was induced by intraluminal occlusion of the left middle cerebral artery (MCA) for 60 min, as previously described (Song et al. 2021). Briefly, mice were anesthetized with isoflurane at an induction rate of 2.5–3.5% and maintained at 1.5%. A midline pre‐tracheal incision was made to expose the common carotid artery (CCA). The CCA and external carotid artery were ligated, and the internal carotid artery was clamped. To block the blood flow in the MCA, a cut was made in the ECA, and a rubber silicon‐coated monofilament suture (size 6–0, diameter 0.09–0.11 mm, length 13 mm; diameter with coating 0.21 ± 0.02 mm; coating length 5 mm) was introduced, advanced along the ICA for 8–9 mm from the carotid artery bifurcation. For reperfusion, the suture was withdrawn 60 min after the time of insertion. Cranial and body temperatures were maintained at 36.5°C ± 0.5°C throughout the surgery with a temperature regulated heating pad. The mice received a pre‐surgical numbing agent application of 0.25% bupivacaine and post‐surgical analgesic right after the procedure and at 1 day post‐surgery by subcutaneous injections of 1 mg/kg ketoprofen in PBS.

### 2.4 Regional Cerebral Blood Flow (rCBF) Measurement

Regional cerebral blood flow measurement of MCA regions was performed using a two‐dimensional laser speckle contrast analysis system (PeriCam PSI High Resolution with PIMSoft; Perimed, Järfälla, Sweden) as described previously (Song et al. 2021). Mice were anesthetized with isoflurane and maintained at physiological body temperature as previously described. The head of the animal was secured in a stereotactic frame (David Kopf Instruments, Tujunga, CA), a midline incision was made by cutting the scalp, and the skull surface was cleaned with sterile normal saline. A charged‐coupled device camera was placed 10 cm above the skull using a Pericam PSI System, and blood perfusion images were taken 5 min prior to transient middle cerebral artery occlusion (tMCAO), 5 min after tMCAO, and 1, 2, and 3 days post‐tMCAO/reperfusion. Raw speckle images were taken in a 1.6 cm × 1.4 cm field (at 19 frames/s). Fifty‐seven frames (with the resolution of 0.02 mm^3^) of consecutive images at each time point per animal were averaged for analysis using equal‐sized, oval‐shaped regions of interest (ROI) covering the frontal and parietal bone plates of the ipsilateral (IL) and contralateral (CL) hemispheres. Cerebral blood flow is expressed in arbitrary units (perfusion units). The CBF measurement method has been validated in our previous studies (Begum et al. 2018; Song et al. 2021). Four animals were excluded from the study due to unanticipated issues: three animals did not survive during the immediate post‐surgical period (two *Nbce1* ^iΔAstro^ and one WT); one animal was excluded due to the drop in blood flow during tMCAO reaching 50% of baseline, which was deemed insufficient. Only mice with a CBF reduction of 65% or more relative to baseline were included.

### 2.5 T2 Magnetic Resonance Imaging (MRI) of Stroke Volume

In vivo T2 MRI was employed to measure infarct volume and hemisphere swelling 3 days post‐tMCAO. WT and *Nbce1* ^iΔAstro^ animals were anesthetized with isoflurane prior to MRI scanning. Respiration and temperature were monitored continuously, and temperature was maintained with a warm heating system (SA instruments, Stony Brook, NY, USA). MRI was performed on a 9.4 T/30‐cm Bruker AVIII HD spectrometer equipped with a 12 cm high‐performance gradient set running ParaVision 6.0.1 (Bruker, Billerica, MA, USA). Mice were placed in the MRI scanner, and following pilot scans, T2‐weighted images (T2WI) were acquired using a Rapid Acquisition with Relaxation Enhancement (RARE) sequence as described previously (Yuan et al. 2023). Infarction volume was corrected for brain edema with the following calculation: Corrected infarct volume = CL hemisphere volume − (IL hemisphere vol‐measured infarct vol). The volumetric analysis was performed using DSI Studio (http://dsistudio.labsolver.org/).

### 2.6 Neurological Function Tests

Behavioral testing was conducted in a blinded manner to evaluate the motor, memory, and sensory function of mice. The baseline motor function of naïve animals was assessed via open field and Y‐maze tests. Post‐stroke sensorimotor function was assessed by foot fault and adhesive tape removal tests performed during the first 3 or 5 days after the tMCAO surgery. Neurological scores were collected through 3 or 5 days after the tMCAO.

#### 2.6.1 Open Field

This test was used to determine gross motor activity and anxiety levels, as previously described (Metwally et al. 2023). Spontaneous naïve mouse activity in Open Field chambers (50 cm × 50 cm × 50 cm) was recorded by Fusion software (Omnitech Electronics Inc.) for 1 h. During this time, all their horizontal and vertical movements, the time spent in the marginal (peripheral) and center zones, and the total distance traveled were recorded using an overhead video tracking system.

#### 2.6.2 Y‐Maze Spontaneous Alternation Test

This test is utilized to evaluate working memory as previously described (Metwally et al. 2023). Naïve mice were placed into a Y‐maze with three equal‐length arms, and spontaneous movements of the mice are tracked using overhead video motion tracking software (Noldus Ethovision XT, version 14). An automated Sequence Analysis Tool macro in Excel was employed to analyze a sequential list of arm entries. Spontaneous alternation was manually counted only when mice entered each of the three different arms sequentially without a predetermined order.

#### 2.6.3 Y‐Maze Novel Spatial Recognition (NSR) Test

This test is utilized to evaluate spatial memory as previously described (Metwally et al. 2023). Naïve mice were placed into a Y‐maze with three equal‐length arms and one arm blocked. After 10 min of familiarization, the mice returned to their home cage. After 5 min, the mice returned to the Y‐maze and were allowed to explore all three arms. Video motion tracking software (Noldus Ethovision XT, version 14) recorded the number of entries into each and the duration in arms. To assess the mice's preference for the novel arm, the differentiation index (DI) was calculated as: (T_novel_ − T_familiar_)/T_total_, and the recognition index (RI) was calculated as: T_novel_/T_total_.

#### 2.6.4 Foot Fault Test

Foot fault testing to detect motor function was conducted once daily for 3 or 5 days preceding tMCAO surgery and for 3 days after surgery, as previously described (Metwally et al. 2023). Mice were placed on a stainless‐steel grid floor (20 cm × 40 cm with a mesh size of 4 cm^2^) and monitored until the test subject reached 50 steps. Movements of the forelimb contralateral to the injured hemisphere are documented: foot fault error was counted when the forelimb fell into the space between the mesh. The final data is obtained by calculating the percentage of unsuccessful steps out of the total steps taken.

#### 2.6.5 Adhesive Sensation and Removal Tests

The adhesive sensation and removal tests were performed to evaluate somatosensory function once daily for 3 or 5 days preceding tMCAO surgery and for 3 days after surgery as previously described (Begum et al. 2018). A piece of adhesive tape measuring (4 mm × 3 mm) was placed in the exact center of the contralateral (right) paw with the aid of forceps. The time at which the tape makes initial contact with the paw was recorded as the sensation time, and the time when the mouse removes the tape from its paw is documented as the removal time. The test was terminated either when the tape was removed from the paw or after 2 min.

#### 2.6.6 Neuroscore

The neurological deficit grading system was used to evaluate neurological deficit at 1–5 days after t‐MCAO as described previously (Begum et al. 2018). Scoring criteria were as follows: 0 = Normal neurological function, no neurological deficit, 1 = continuous flexion of the contralateral forelimb, 2 = decreased resistance against lateral push, 3 = spontaneously or when lifted by the tail exhibits unilateral circling movement, 4 = no response to external stimulation or stroke‐related death. Neuroscoring was conducted 1 day before the surgery, 1 h following tMCAO surgery (after the reperfusion) and then retested daily for 3 or 5 days after the reperfusion.

### 2.7 2,3,5‐Triphenyl Tetrazolium Chloride (TTC) Staining

Infarct volume was determined by TTC staining at 3 days after tMCAO surgery, as described before (Bhuiyan et al. 2017). The brains were sectioned at a thickness of 2 mm. Infarction volume was corrected for brain edema with the following calculation: Corrected infarct volume = CL hemisphere volume − (IL hemisphere vol‐measured infarct vol) and quantified using ImageJ software. The extent of hemispheric swelling was calculated using the equation: (Volume of IL hemisphere − Volume of CL hemisphere)/Volume of CL hemisphere (Bhuiyan et al. 2017).

### 2.8 Evans Blue Staining

BBB leakage was assessed using the Evans blue extravasation assay as described previously (Begum et al. 2018). Briefly, mice were injected with 2% Evans blue solution in phosphate buffered saline (PBS) (10 mL/kg; i.p.) 3 days post‐tMCAO. Two hours after injection, mice were euthanized by CO_2_ overdose, and brains were collected following cardiac perfusion with PBS and 4% paraformaldehyde (PFA) in PBS. The brains were then sectioned and stained with *Lycopersicon esculentum* (Tomato) Lectin (LEL, TL), DyLight 488 in PBS overnight, followed by a 15 min incubation with DAPI. EB fluorescence signals were excited at 635 nm, with emission detected at 680 nm. Fluorescent images were captured using an Olympus IX83 Microscope System with a 4× objective or a FV1000 laser scanning confocal system with a 20× objective.

### 2.9 Immunofluorescence Staining and Imaging

Staining was performed in free‐floating microtome sections as described before (Begum et al. 2018). Briefly, mice were deeply anesthetized and transcardially perfused with 4% PFA in 0.1 M tris buffered saline (TBS). Brains were postfixed in 4% PFA for 24 h, followed by incubation in 30% sucrose for 12–16 h at 4°C. Using a microtome (Leica VT1200S), 25 μm thick coronal tissue sections were prepared (−0.38 mm bregma). Brain sections were washed with TBS and incubated with blocking solution (10% normal goat serum (NGS), 5% BSA and 0.5% Triton X‐100 in 0.01 M TBS) for 1 h at room temperature (RT) followed by incubation with primary antibodies. All the primary antibodies were diluted in the blocking solution containing TBS^++^ solution (3% NGS and 0.3% Triton X‐100 in TBS) and incubated with brain sections overnight at 4°C. Antibodies and their concentrations used are listed in Table S1. Astrocytes were labeled with anti‐GFAP antibodies. Anti‐AQP4 antibodies were used to visualize astrocyte endfeet, while blood vessels were identified using anti‐CD31 (PECAM‐1), *Lycopersicon esculentum* (Tomato) Lectin (LEL, TL), or lectin DyLight 488. Following primary antibody incubation, brain sections were incubated for 1 h at RT with appropriate secondary antibodies (all diluted 1:200). Nuclei were stained with DAPI (1:1000 in blocking solution) or TO‐PRO‐3 Iodide (1:1000). For negative controls, brain sections were stained with secondary antibodies only (Figure S1A,B). Fluorescent images were acquired using the following microscopes: Olympus IX83 inverted microscope with 4× or 10× objectives, Olympus IX81 inverted microscope with a FV1000 laser scanning confocal system and a 40×/60× oil immersion objective, Leica TCS SP5 confocal system with a 63× oil immersion objective, or NIKON A1R confocal microscope with 40×, 60×, or 100× oil immersion objectives. The images were captured at 1024 × 1024‐pixel resolution (0.103 μm/pixel). All imaging and subsequent analyses were performed in a blinded manner.

### 2.10 DiOlistic Labeling

#### 2.10.1 Tissue Sectioning With Vibratome

Tissue sectioning was performed as described previously (Waxman et al. 2023). Briefly, at 3 days post stroke, mice were perfused with PBS followed by 4% PFA. Brains were harvested and post‐fixed in 4% PFA for 6 h, then embedded in low gelling temperature agarose and mounted onto the vibratome stage (Leica, VT1200S) using cyanoacrylate glue. The stage was filled with ice‐cold PBS, and coronal slices were cut at 150 μm thickness. To prevent tissue damage, blade advancement speed was set to a slow pace (0.01–0.05 mm/s). Slices were collected using a paintbrush and stored temporarily in ice‐cold PBS.

#### 2.10.2 Multicolor DiOlistics Bullet Preparation

Multicolored DiOlistic bullets were prepared as described previously (Waxman et al. 2023). Briefly, 70 mg of 1.0 μm gold microparticles were equally distributed into seven 1.5 mL Eppendorf tubes, each receiving 100 μL of ethanol. Stock dye solutions were prepared by dissolving 4 mg each of dye—DiI, DiO, and DiD—in 500 μL of ethanol. The dye solution was then added to the gold particle‐containing tubes in all possible combinations (only DiO, only DiD, only DiI, DiO‐DiD, DiO‐DiI, DiD‐DiI, and DiO‐DiD‐DiI), ensuring each tube received an equal amount of dye. The dye‐gold microcarriers were gently pipetted and vortexed for ~30 s. The gold‐dye combination was then pipetted onto glass slides and air dried at RT for 20 min. Dried microcarriers were scraped off the slides using a razor blade and resuspended in 3 mL of deionized water and filtered through a 20 μm filters to remove large clumps. Filtered microcarriers were loaded into Tefzel tubing using a tubing preparation station. The loaded tubing was left undisturbed for 10 min to allow the microcarriers to settle at the bottom. Water was then slowly drawn out of the tubing, leaving the sedimented dye‐coated gold behind. To remove any remaining liquid, nitrogen gas was passed through the tubing for 1 h at 0.2 L per minute. Tubing was then cut into 1.3 cm segments to produce gene gun bullets. The bullets were stored at RT, protected from light, with a desiccant, for up to 6 months.

#### 2.10.3 DiOlistic Cell Labeling With Gene Gun

The Helios Gene Gun was loaded with prepared bullets and connected to a pressurized nitrogen tank set to 200 PSI to deliver dye‐coated microcarriers into 150 μm‐thick brain slices. Prior to labeling, brain sections were placed in a chamber, and excess PBS was removed using a Kimwipe. A 20 μm filter was positioned over the section, ensuring proper coverage. The gene gun was then aligned directly over the filter and fired, with each slice receiving two bullets. Labeled brain slices were then transferred back into PBS before being mounted onto glass slides using a water‐based mounting medium.

### 2.11 IMARIS 3D Cellular Image Reconstruction

#### 2.11.1 Quantification of AQP4/NBCe1 Signals in GFAP ^+^ Astrocytes

AQP4 signal quantification in GFAP^+^ astrocyte cell volume was performed using the surface component in IMARIS 10.0.1. The confocal Z‐Stack images were imported into IMARIS and converted into 3D surface renderings. A new surface was generated with the object‐object statistics option enabled in the algorithm settings. Surface parameters were adjusted to label AQP4. A voxel‐based filter was applied to remove small staining artifacts, with no further surface classification. Subsequently, a second surface was created, and surface parameters were adjusted to label GFAP^+^ astrocytes. The results were recorded in the detailed section of the statistics tab. An overlapped volume ratio of AQP4 and GFAP^+^ surfaces were analyzed and a comparison analysis between WT and *Nbce1* ^iΔAstro^ was performed.

#### 2.11.2 GFAP ^+^ Immunostaining and DiOlistic Labeling Analysis for Assessing Astrocyte Volume

GFAP^+^ astrocyte cell volume was performed using the surface component in IMARIS 10.0.1. The confocal Z‐Stack images were imported into IMARIS and converted to 3D surface renderings. A new surface was generated with the object‐object statistics option enabled in the algorithm settings. Surface parameters were adjusted to label astrocytes. A voxel‐based filter was applied to remove small staining artifacts, with no further surface classification. The results were recorded in the detailed section of the statistics tab. Astrocyte volumes were extracted, and a comparison analysis between WT and *Nbce1* ^iΔAstro^ was performed.

### 2.12 RNA In Situ Hybridization and Image Quantification

PFA‐fixed brains were frozen in Tissue‐Tek OCT at −80°C in cryo molds. The frozen blocks were sectioned (16 μm thick sections) using a cryostat (Leica, Cryostar NX50) and placed onto Superfrost Plus Microscope Slides and stored at −80°C until use. RNA in situ hybridization was performed using the ACDBio RNAscope Multiplex Fluorescent Reagent Kit (v2) according to the manufacturers instructions. Briefly, slides were washed in 1× PBS (phosphate buffered saline) for 5 min, followed by baking them for 30 min at 60°C. Slides were then post‐fixed by immersing them in prechilled 4% PFA for 15 min. Following a rinse with PBS, the slides were dehydrated with ethanol at 50%, 75%, and 100% (2×) for 5 min at RT. Samples were then air dried for 5 min at RT and incubated with 3–4 drops of H_2_O_2_ for 10 min at RT, followed by 2–3 washes with ultra pure water (Milli‐Q, Millipore). Target retrieval was then performed by immersing the samples in freshly prepared 1× target retrieval reagent in a beaker maintained at 99°C–100°C contained in a steamer for 15–30 min. The samples were then washed in ultra pure water at RT and immersed in 100% EtOH for 3 min, followed by drying the slides in an incubator at 60°C. Lipid barriers were drawn around the brain sample with an Immedge pen. Samples were then incubated in Protease III for 30 min in a HybEZ Oven at 40°C. The proteases were washed twice with ultra pure water, and probe hybridization was carried out at 40°C for 2 h with mouse *Nbce1* in C2 and a 3‐plex negative control probe in C3. The samples were then washed with wash buffer twice for 2 min and then amplified according to the v2 protocol. TSA‐cy3 (1:1000) (Perkin Elmer FP1170) was added to the samples and incubated for 30 min at 40°C, followed by incubation with Multiplex FL v2 HRP blocker for 15 min at 40°C, followed by washing twice in 1× wash buffer for 2 min at RT. Samples were then immediately used for immunofluorescence staining using anti‐GFAP antibodies following the procedure described in the previous section. Negative control images stained with GFAP antibodies and a 3‐plex negative control probe are included in Figure S2. Brain sections were mounted with ProLong Gold Antifade Mountant, cover‐slipped, and imaged using an Olympus FV3000 confocal laser scanning microscope.

RNAscope image quantification was done using Qupath (v0.4.3) (Bankhead et al. 2017). Individual nuclei and GFAP‐positive astrocytes were detected based on DAPI and Alexa 488 signal. Duplicate channel training images were then generated to train the object classifier in each channel. Object classifiers were then loaded onto the merged images to generate cell counts. After the classification, the NBCe1 signal was detected based on the Alexa 546 signal by using a subcellular detection tool.

### 2.13 Immunoblotting

Immunoblotting was performed as described previously (Liu et al. 2022). Briefly, brain tissue or isolated astrocytes from CL and IL hemispheres of WT and *Nbce1* ^iΔAstro^ brains at 3 days post‐tMCAO were homogenized in RIPA lysis buffer (ThermoFischer Scientific, Rockford, IL) containing a protease and phosphatase inhibitor mixture (Roche) and centrifuged at 4°C for 30 min at 14,000 × g. The supernatant was transferred to a fresh tube and concentration was determined by a BCA kit (Thermo Scientific). An equal amount of proteins was separated by 4%–15% SDS‐PAGE and electro transferred onto a PVDF membrane (0.2 μm). After blocking with 5% BSA in TBS‐Tween, the membrane was incubated O/N at 4°C with primary antibodies. Following primary antibody incubation, the blots were washed and incubated with appropriate secondary antibody conjugates with horseradish peroxidase for 60 min at RT. Protein bands were imaged using the ChemiDoc MP Imaging System, using SuperSignal **West** Pico PLUS Chemiluminescent Substrate (Fisher Scientific, USA). The protein expression levels were calculated by measuring the intensities of the bands using Image Lab Software (BioRad, USA) and normalized against the total protein values.

### 2.14 Cytokine Protein Array

The cytokine and chemokine levels in post‐tMCAO brains were analyzed by a membrane‐based sandwich immunoassay using the Proteome Profiler Mouse Cytokine Array Kit, Panel A (R&D Systems, Minneapolis, MN) according to the manufacturer's instructions. Briefly, the IL and CL brain hemispheres were homogenized in RIPA buffer (2 mL/100 mg) and centrifuged for 20 min (4°C) at 14,000 rpm (revolutions per minute) to collect the supernatants. IL and CL brain regions from five mice were pooled separately. Protein concentration of samples was quantified via BCA assay, and 40 μg from each pooled sample (total 200 μg per group) was diluted in Array Buffer 6 to a final volume of 100 μL. The samples were incubated with the cytokine array membrane according to the manufacturer's protocol. The signals were acquired using the ChemiDoc MP Imaging System using SuperSignal West Pico PLUS Chemiluminescent Substrate. The pixel intensities were measured and analyzed using the Image Lab Software (BioRad, USA). Values from duplicate spots were averaged, and the relative signal was calculated.

### 2.15 Flow Cytometric Measurement of Astrocytic Intracellular pH

Flow cytometry was conducted to investigate the differences in the intracellular pH of CL and IL hemispheres of WT and *Nbce1* ^iΔAstro^ brains 3 days post‐tMCAO. Animals were transcardially perfused with PBS, and brains were harvested. Single‐cell suspensions from the IL and CL hemispheres of WT and *Nbce1* ^iΔAstro^ brains were prepared using the adult brain dissociation kit, as described previously (Song et al. 2021). Briefly, following enzymatic and mechanical digestion of the hemispheres, the homogenate was filtered through a 70 μm filter and subjected to debris removal to eliminate myelin. The isolated cell pellet was resuspended in Hank's balanced salt solution (HBSS) supplemented with 1% fetal bovine serum (FBS). The cells were stained with fluorescein isothiocyanate‐conjugated antibody against astrocyte antigen 2 (FITC‐ACSA2^+^) (1:400 in HBSS plus 1% FBS) at 4°C for 20 min. Cells were washed and incubated for 30 min at 37°C with PE fluorophore‐conjugated pHRODO (5 μM or 1: 1000 stock solution), which has stronger fluorescence with increased acidity. The stained cells were passed through the BD LSRFortessa Cell Analyzer (BD Biosciences) using BD FACSDiva Software. FlowJo V8 software was used to gate out debris using a forward scatter area versus side scatter area (FSC‐A vs. SSC‐A) plot, identify singlets through a side scatter area versus side scatter width (SSC‐A vs. SSC‐W) plot, and select FITC^+^ singlets using a FITC histogram, which represents ASCA2^+^ astrocytes. Among these ASCA2^+^ astrocytes that absorbed the PE pHRODO dye, the mean fluorescent intensity (MFI) for PE pHRODO red was calculated (PE histogram). The MFIs of the IL hemispheres were normalized to the CL hemispheres for both WT and *Nbce1* ^iΔAstro^ mice.

### 2.16 Statistical Analysis

All experimenters were blinded to group allocation. Litters were housed in mixed groups after i.p. injection with Tam and also upon surgery. Experimental animals were age‐ and sex‐matched. A total of 130 mice (2–3‐month‐old; males: 27 ± 3 g, females: 22.5 ± 3 g) were used, with an overall mortality rate of 22.7% at 5 days post‐stroke and 21.5% at 3 days post‐stroke. The mortality rate was 23.1% in WT stroke mice (22.6% in F, 23.7% in M) and 20% in *Nbce1* ^iΔAstro^ stroke mice (19.8% M, 20.2% F) at 3 days post‐stroke. Five mice were excluded from the study due to the absence of stroke symptoms. Mice were administered tamoxifen at 2–3 months of age, and tMCAO surgeries were performed at 4–6 months of age. Behavioral studies and immunocytochemical analyses were performed on the same cohort, with brain collected at different time points. Group sizes were *N* = 4–9 for behavioral tests and *N* = 4–7 for immunostaining and MRI. Data are presented as mean ± SEM/SD, and all data were tested for normal distribution using the Kolmogorov–Smirnov test. For comparing two conditions, a two‐tailed Student's *t*‐test with 95% confidence was employed. A one‐way or two‐way analysis of variance was used to compare three or more conditions, followed by Sidak's multiple comparisons test. Statistical significance was considered at a *p* < 0.05 (Prism 10, GraphPad, San Diego, CA, USA). Non‐normally distributed data were analyzed using a two‐tailed unpaired Mann–Whitney *U*‐test with a confidence level of 95% or other appropriate alternative tests according to the data. All data were included unless excluded by outlier analysis suggests otherwise.

## 3 Results

### 3.1 Generation and Characterization of *Nbce1* ^iΔAstro^ Mice

**FIGURE 1:**
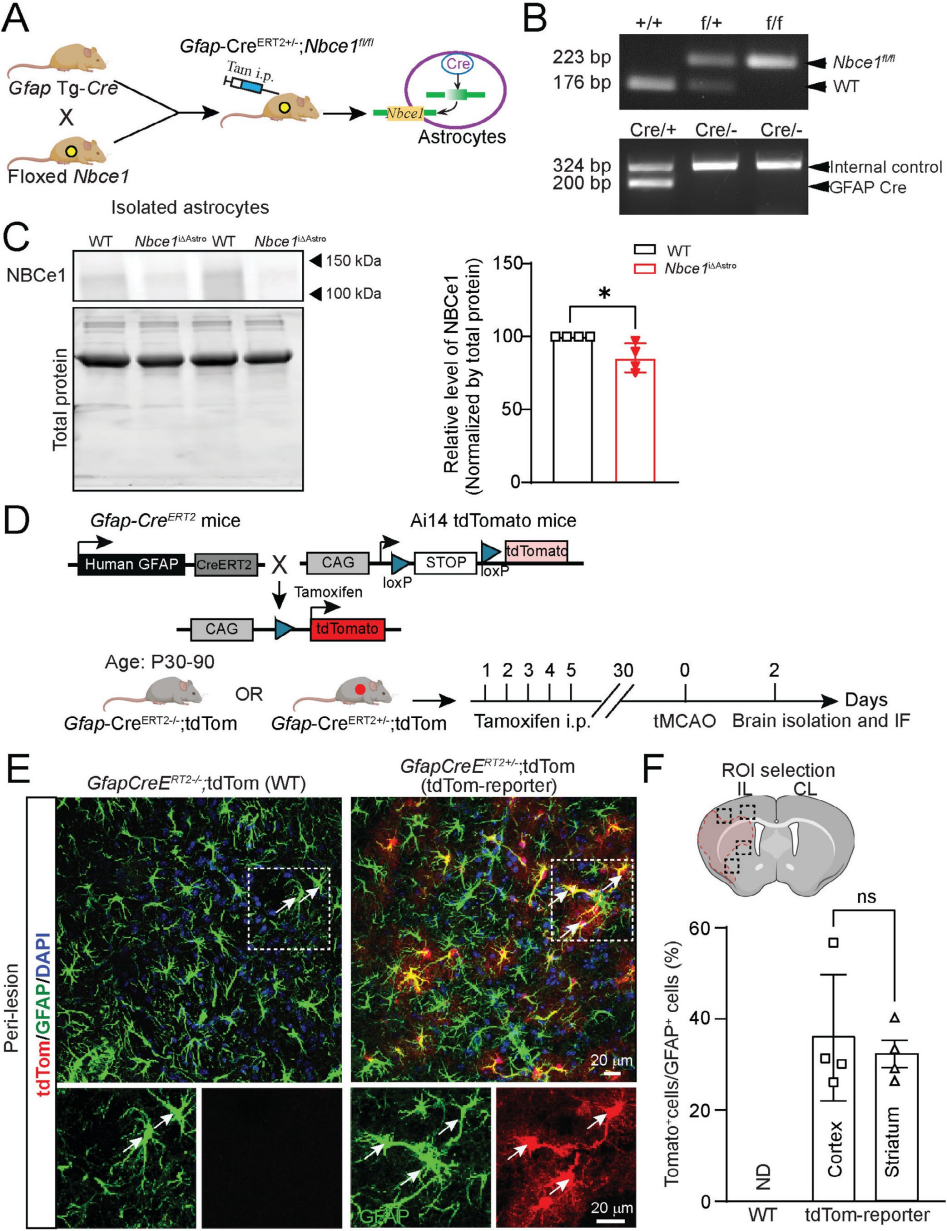
Generation and characterization of *Nbce1* ^iΔAstro^ mice. (A) Breeding scheme and generation of astrocyte‐specific *Nbce1* knockout mice. (B) Representative PCR analysis of ear biopsy DNA for identification of Cre recombinase and *Nbce1* floxed allele. (C) Representative western blotting image and quantification of NBCe1 protein expression in astrocytes isolated from WT and astrocyte‐specific *Nbce1* knockout mice. Data are expressed as relative change to WT control. Data are mean ± SD, via unpaired *t*‐test; *n* = 4; *p < 0.05 vs. WT. (D) Genetic map and breeding scheme of *GfapCre* ^ERT2+/−^ and Ai14 tdTomato reporter mouse line. (E) Representative confocal images of GFAP immunostaining and tdTom transgene expression in peri‐lesional cortex of WT and *GfapCre* ^+/−^; tdTom reporter mice at 3 days post‐stroke. (F) Quantification of GFAP‐expressing cells recombined as evaluated by tdTom co‐expression. Data are mean ± SD, *n* = 4; ns = not significant via unpaired *t* test.

We utilized a previously validated *Nbce1 flox/flox* (*Nbce1* ^fl/fl^) mouse line with LoxP sites targeting exon 12 of the *Slc4a4* gene6 (Brown et al. 2021; Vairamani et al. 2018). We generated astrocyte‐selective *Nbce1* conditional knockout (*Nbce1* ^iΔAstro^) mice by injecting tamoxifen (Tam; 75 mg/kg i.p. for 5 days) into *Nbce1* ^f/f^ mice that have been crossed with inducible *Gfap‐Cre* ^ERT2+/−^ (Ganat et al. 2006), as illustrated in Figure 1A. Tam‐injected *Gfap‐Cre* ^ERT2−/−^; *Nbce1* ^fl/fl^ were used as WT controls. The WT and floxed alleles were identified by a 178 bp and 223 bp DNA product (Figure 1B), respectively, via PCR analysis as described earlier (Brown et al. 2021). Behavioral studies were conducted to assess basic parameters such as motor skills, stress, learning, and memory in naïve WT and *Nbce1* ^iΔAstro^ mice. No significant changes were seen in motor function or learning and memory tests (Figures S3 and S4). However, in the open field test, *Nbce1* ^iΔAstro^ naïve mice exhibited significantly increased vertical activity (Figure S4; *p* < 0.05), a behavioral pattern indicative of enhanced exploratory drive and environmental curiosity (Concetti et al. 2024). A significant reduction in NBCe1 protein expression was detected in ACSA2^+^ astrocytes isolated from naïve *Nbce1* ^iΔAstro^ mice, compared to WT mice (14.8% ± 4.9%; *p* = 0.029; Figure 1C) with Western blot analysis.

Next, the extent of Cre‐mediated recombination in *GfapCre* ^ERT2+/−^ line induced by Tam injection was assessed by crossing *GfapCre* ^ERT2+/−^ mice with Ai14 Cre‐dependent tdTomato reporter mice to generate *GfapCre* ^ERT2+/−^; Ai14‐tdTom mice (tdTom reporter mice) (Figure 1D). Post‐Tam administration, WT and tdTom reporter mice underwent 60‐min tMCAO to induce ischemic stroke and astrogliosis with increased GFAP expression in reactive astrocytes at 3 days post‐stroke. As shown in Figure 1E, no tdTom activation was detected in GFAP^+^ reactive astrocytes in the peri‐lesion cortical tissues of WT stroke brains (*GfapCre* ^ERT2−/−^; tdTom). In contrast, tdTom reporter stroke mice (*GfapCre* ^ERT2+/−^; tdTom) displayed selective tdTom expression in GFAP^+^ reactive astrocytes peri‐lesion areas (32.2% ± 3% in the cortex and 35.9% ± 6.9% in the striatum; Figure 1F), indicating effective Cre recombination as previously reported (Park et al. 2018).

### 3.2 *Nbce1* ^iΔAstro^ Mice Displayed Reduced Infarct Volume and Improved Sensorimotor Functions After Ischemic Stroke

**FIGURE 2:**
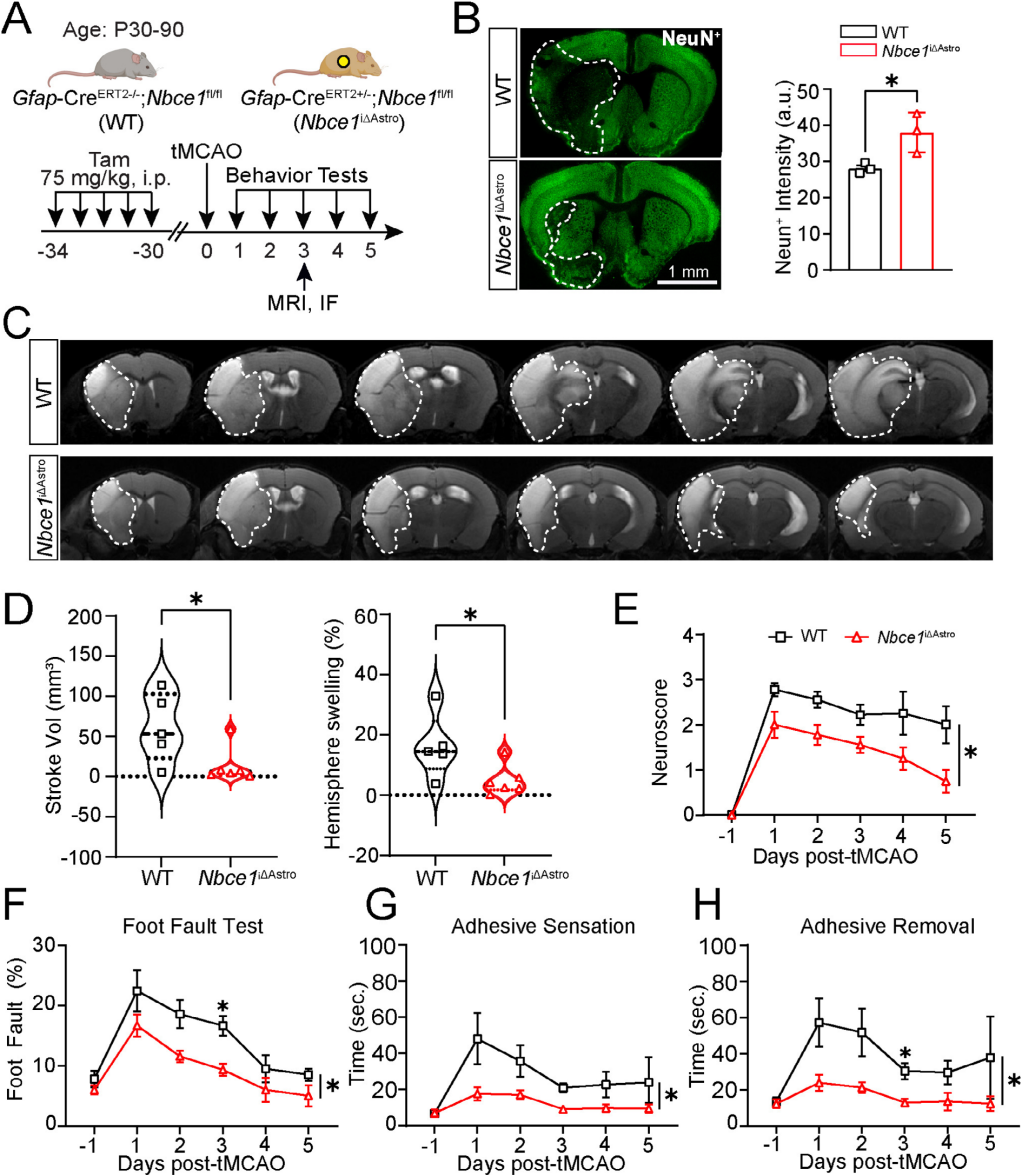
*Nbce1* deficiency in astrocytes reduced infarct volume and improved sensorimotor functions in stroke mice. (A) Schematic of experimental protocol and timeline. (B) Representative NeuN^+^ staining images and summary of neurodegeneration in WT or *Nbce1* ^iΔAstro^ brains at 3 days post stroke. Data are mean ± SD. *n* = 3; *p < 0.05 vs. WT via unpaired *t*‐test. (C) Representative T2‐weighted MRI brain images of WT and *Nbce1* ^iΔAstro^ brains. (D) Quantitative analysis of stroke and hemispheric swelling volume. Data are presented as violin plots. *n* = 5–6; *p < 0.05 vs. WT via unpaired *t*‐test. (E) Neuroscore summary. Data are mean ± SEM. *n* = 4–9; *p < 0.05 vs. WT via two‐way ANOVA followed by Sidak's multiple comparisons. (F–H) Foot fault and adhesive sensation and removal tests prior to tMCAO surgery (−1, baseline) and at 1–5 days post‐tMCAO surgery. Data are mean ± SEM. *n* = 9; *p < 0.05 vs. WT via two‐way ANOVA followed by Sidak's multiple comparisons.

We next assessed the impact of astrocytic *Nbce1* gene deletion on ischemic stroke outcomes (Figure 2A). Evaluation of infarct size in WT and *Nbce1* ^iΔAstro^ mice was performed either with T2WI or by TTC staining at 3 days post‐stroke. T2WI sequence parameters are listed in Table S2 in the supplemental file. The WT stroke mice exhibited larger infarct volume (60.8% ± 19.1%) and hemispheric swelling (16.2% ± 4.6%) on MRI (Figures 2C and S5). In contrast, significantly reduced stroke volume (13.6% ± 9.1%) and hemispheric swelling (4.8% ± 2%) were detected in *Nbce1* ^iΔAstro^ mice at 3 days post‐stroke (*p* < 0.05, Figure 2C,D). TTC staining of brain sections at 1, 3, 5, and 7 mm from the frontal pole also revealed larger infarcts and hemispheric swelling in WT mice, whereas *Nbce1* ^iΔAstro^ mice exhibited significantly reduced infarct and swelling volume (*p* < 0.05, Figure S6). Notably, there was a significant reduction in the loss of NeuN^+^ neurons in the *Nbce1* ^iΔAstro^ *stroke* brains (*p* < 0.05, Figure 2B). In the neurological function tests, *Nbce1* ^iΔAstro^ mice exhibited reduced neurological deficits compared to the WT stroke mice, including less impairment in the modified neurological severity score (mNSS) and foot‐fault test (Figure 2E,F). The adhesive removal test revealed that when compared with the WT stroke mice, the *Nbce1* ^iΔAstro^ stroke mice displayed faster limb sensorimotor function at 3 days post‐stroke (*p* < 0.05, Figure 2G,H). Together, these data collectively demonstrated that astrocytic NBCe1 protein is negatively involved in ischemic stroke brain damage.

### 3.3 NBCe1 Expression Is Upregulated in GFAP ^+^ Reactive Astrocytes Within the Peri‐Lesion Areas of WT Stroke Brains

**FIGURE 3:**
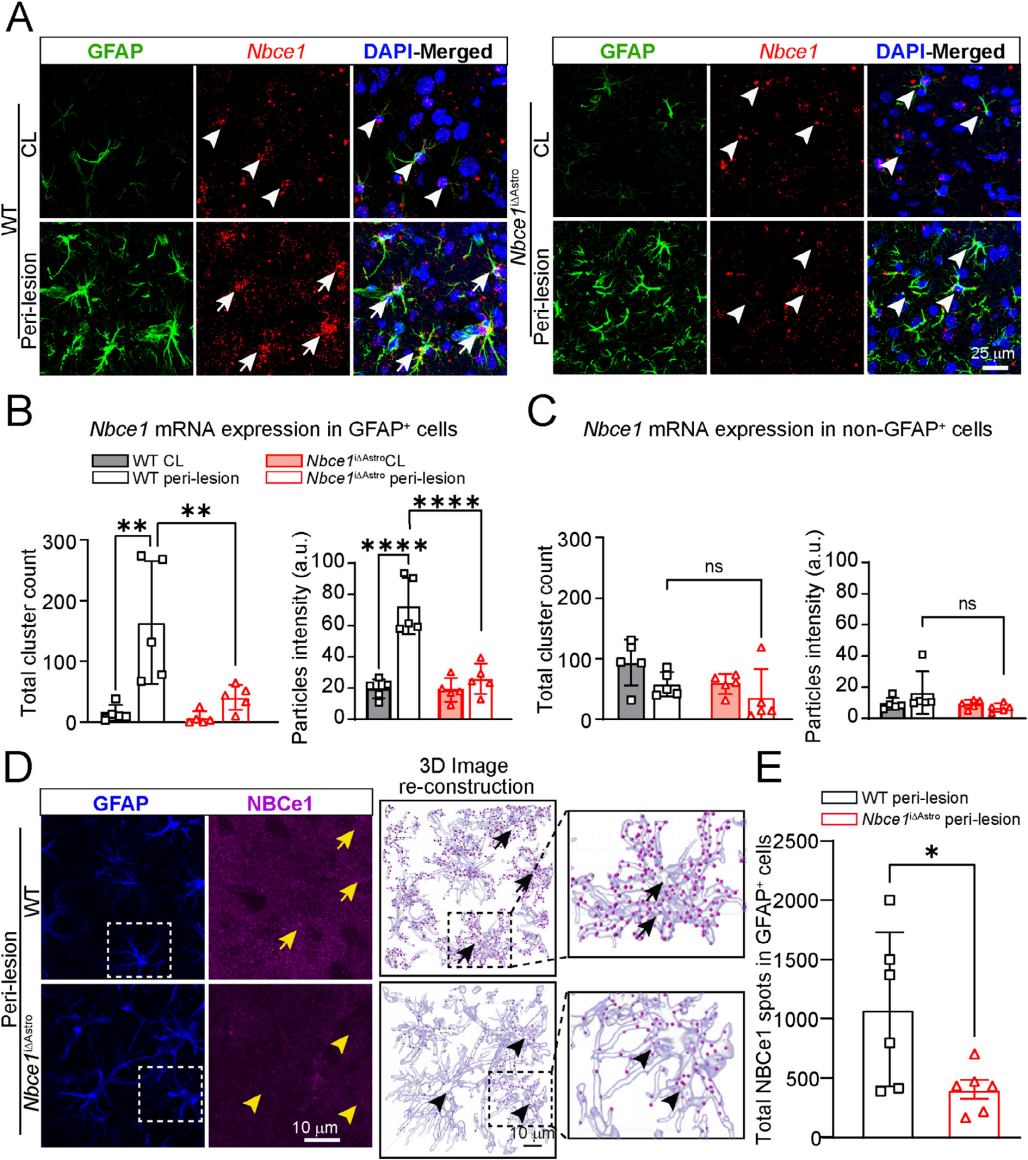
NBCe1 expression is upregulated in GFAP^+^ reactive astrocytes following ischemic stroke. (A) Representative confocal images showing dual RNAscope F‐ISH and immunofluorescence signals using *Nbce1* probes and GFAP antibodies in WT and *Nbce1* ^iΔAstro^ mice in the peri‐lesion areas at 3 days post stroke. Arrows: High expression; Arrow heads: Low expression. (B, C) Quantification of RNAscope signal as *Nbce1* transcript clusters in GFAP^+^ and non‐GFAP^+^ cells. Data are mean ± SD, *n* = 5–6. **p < 0.01, ****p < 0.001; versus the indicated group via two‐way ANOVA followed by Sidak's multiple comparisons. (D) Representative confocal images showing NBCe1 protein expression in IL peri‐lesion areas of WT and *Nbce1* ^iΔAstro^ brains at 3 days post stroke. (E) Quantification of NBCe1^+^ puncta within GFAP^+^ astrocytes. Data are mean ± SD, *n* = 5–6. *p < 0.05 versus the indicated group via unpaired *t*‐test.

We speculate that the NBCe1 protein is selectively upregulated in reactive astrocytes after ischemic stroke, potentially exerting detrimental effects on neuronal survival and recovery processes. To examine changes in NBCe1 protein and mRNA expression in stroke brains, we used multiplexed fluorescence in situ hybridization (FISH, by RNAscope) combined with immunofluorescence to map *Nbce1* transcripts in WT and *Nbce1* ^iΔAstro^ brains in astrocytes at 3 days post‐stroke. As shown in Figure 3A, low levels of *Nbce1* transcripts were detected in GFAP^+^ astrocytes within the cortex and striatum of CL hemispheres of WT and *Nbce1* ^iΔAstro^ brains at 3 days post‐stroke. However, in the IL peri‐lesion areas of WT brains, GFAP^+^ astrocytes exhibited increased *Nbce1* expression (Arrows, Figure 3A). This increase was quantified by both *Nbce1* cluster counts and mean fluorescence intensity of *Nbce1* clusters in the cell soma and processes (Figure 3B). In contrast, GFAP^+^ astrocytes in the peri‐lesion areas of *Nbce1* ^iΔAstro^ brains showed significantly reduced *Nbce1* cluster counts (40.5 ± 9.2 vs. 163.8 ± 45.2; *p* = 0.01) and intensity (Arrow heads, Figure 3A,B). No significant changes in *Nbce1* expression were detected in the non‐GFAP^+^ cells in either the CL or IL hemispheres of WT and *Nbce1* ^iΔAstro^ brains, indicating that astrocytes are the predominant cell type responsible for NBCe1‐mediated changes in the brain (Figure 3C). Immunofluorescence analysis revealed increased NBCe1 protein expression in GFAP^+^ reactive astrocytes within the IL peri‐lesion area of WT brains (Arrows, Figure 3D). However, *Nbce1* ^iΔAstro^ brains exhibited reduced NBCe1 protein expression in the GFAP^+^ reactive astrocytes within the same IL peri‐lesion regions (Arrow heads, Figure 3D). Quantification of NBCe1^+^ immunopuncta within the GFAP^+^ soma and processes, performed using 3D rendering spot analysis with IMARIS software, further revealed a significant increase of NBCe1 puncta counts in the reactive astrocytic soma in WT stroke brains (*p* = 0.03; Figure 3D,E). In contrast, *Nbce1* ^iΔAstro^ stroke brains exhibited significantly reduced NBCe1^+^ puncta in GFAP^+^ reactive astrocytes. Analysis of NBCe1 protein expression in the ischemic core at 1‐ and 3‐days post‐stroke revealed a loss of NBCe1^+^ uniform punctate signals seen in healthy CL hemispheres (arrows, Figure S7A). However, we detected clustered NBCe1^+^ immunosignals throughout the ischemic core (arrow heads, Figure S7A) in both WT and *Nbce1* ^iΔAstro^ stroke brains. This pattern likely reflects a combination of necrotic tissue, infiltration of inflammatory cells, and nonspecific antibody accumulation, contributing to clustered signals absent in structurally intact healthy CL regions (Figure S7A,B). Quantification of fluorescence intensity in the core region did not show any significant difference between the WT and the *Nbce1* ^iΔAstro^ stroke brains, indicating localized loss or degradation of NBCe1 protein in the ischemic core. However, immunoblotting analysis failed to detect differential NBCe1 protein expression (at ~130 kDa) in MACS isolated ACSA2^+^ astrocytes from WT and *Nbce1* ^iΔAstro^ stroke brains (Figure S8) or between CL and IL hemispheres. This is likely because spatially restricted upregulation of NBCe1 within peri‐lesional reactive astrocytes was masked by the inclusion of non‐reactive astrocytes in the bulk lysates obtained from the entire ischemic hemisphere, including the infarct core. Taken together, our findings demonstrate that NBCe1 protein expression is upregulated in reactive astrocytes within the peri‐lesional areas of WT brains.

### 3.4 *Nbce1* ^iΔAstro^ Brains Displayed Reduced BBB Damage After Ischemic Stroke

**FIGURE 4:**
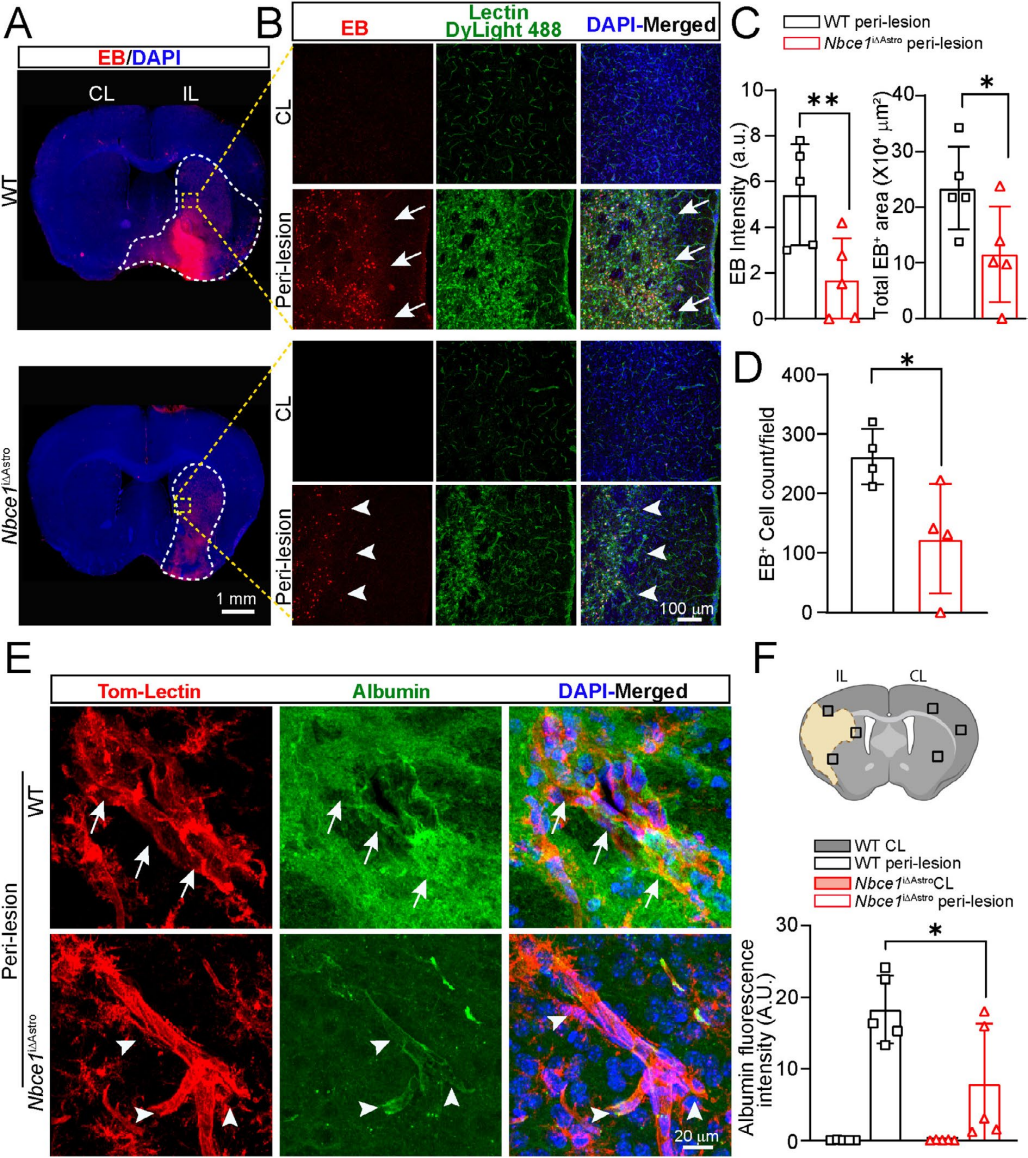
Selective deletion of *Nbce1* in GFAP^+^ astrocytes reduced BBB damage in ischemic stroke brains. (A) Representative low magnification brain map images of EB and DAPI fluorescence in WT and *Nbce1* ^iΔAstro^ brains at 3 days post‐stroke. (B) Representative confocal images of EB and lectin fluorescence in WT and *Nbce1* ^iΔAstro^ stroke brains. Arrows: High Evans Blue (EB) fluorescence. Arrowheads: Low EB fluorescence. (C, D) Quantification of EB dye intensity, penetrated area, and EB^+^ cells in two groups. Data are mean ± SD, *n* = 4–5; *p < 0.05, **p < 0.01 vs. WT IL via unpaired *t* test. (E) Representative confocal Z‐stack images of tom‐Lectin labeled vessels and albumin leakage in the peri‐lesion regions. Arrows: High expression. Arrowheads: Low expression. (F) Schematic of data collection from contralateral and peri‐lesion regions and quantification of albumin fluorescence intensity. Data are mean ± SD, *n* = 5; *p < 0.05 vs. WT IL via unpaired *t* test.

Next, we quantified changes in BBB integrity in WT and *Nbce1* ^iΔAstro^ stroke brains at 3 days post‐stroke using Evans blue dye (EB). EB is a low molecular weight (MW 960 Da) substance that can penetrate the disrupted BBB after systemic administration, and its accumulation in the parenchyma can be quantified through fluorescence imaging (Belayev et al. 1996). EB fluorescence was not detected in the CL hemispheres of WT and *Nbce1* ^iΔAstro^ stroke brains (Figure 4A). However, in the ischemic hemispheres of WT brains, EB fluorescence was found accumulating in the ventromedial striatum and extending into cortical areas (Figure 4A brain maps), indicating significant BBB damage associated with prolonged MCAO (Pan et al. 2017). An increase in EB fluorescence was also detected in the extravascular spaces of the peri‐infarct regions and accumulation in necrotic cells within the infarct area of WT brains (Arrows, Figure 4B). However, in *Nbce1* ^iΔAstro^ stroke brains, a significant reduction in EB extravasation was observed in the IL hemispheres (*p* < 0.05, Figure 4B,C). Moreover, as shown in Figure 4B, in the non‐ischemic CL hemispheres of WT and *Nbce1* ^iΔAstro^ stroke brains, lectin labeling clearly identified the vascular structures, and EB fluorescence was not detected in the brain parenchyma, indicating intact BBB. Quantification of EB positive cells revealed an increase in WT ischemic brains (262 ± 23 cells/field of view) compared to a significantly lower number of EB positive cells in the *Nbce1* ^iΔAstro^ ischemic hemispheres (123 ± 46 cells/field of view; *p* < 0.05) (Figure 4D), indicating reduced BBB damage. To assess the extent of vascular damage, parenchymal infiltration and vascular accumulation of serum albumin, a high molecular weight protein and indicator of BBB damage (Krueger et al. 2019) was evaluated in WT and *Nbce1* ^iΔAstro^ stroke brains. No extravasation of the permeability marker FITC‐albumin was detected in the parenchyma or lectin^+^ vessels in the naïve WT and *Nbce1* ^iΔAstro^ brains (Figure S9). In contrast, the IL hemispheres of WT brains at 3 days post‐stroke showed a pronounced increase in parenchymal albumin extravasation and in the lectin^+^ vessels (Figure 4E). In contrast, *Nbce1* ^iΔAstro^ stroke brains exhibited reduced parenchymal albumin leakage (Figure 4E). Quantification of the stained area revealed significantly elevated extravasation in the ischemic peri‐infarct areas of WT brains compared to the *Nbce1* ^iΔAstro^ stroke brains at 3 days post‐stroke (*p* < 0.05; Figure 4F). Together, these data suggest that deletion of astrocytic *Nbce1* reduces stroke‐induced BBB damage in *Nbce1* ^iΔAstro^ stroke brains.

### 3.5 Selective Deletion of *NBCe1* in GFAP ^+^ Astrocytes Preserved Perivascular AQP4 Distribution and Reduced Its Parenchymal Expression After Ischemic Stroke

**FIGURE 5:**
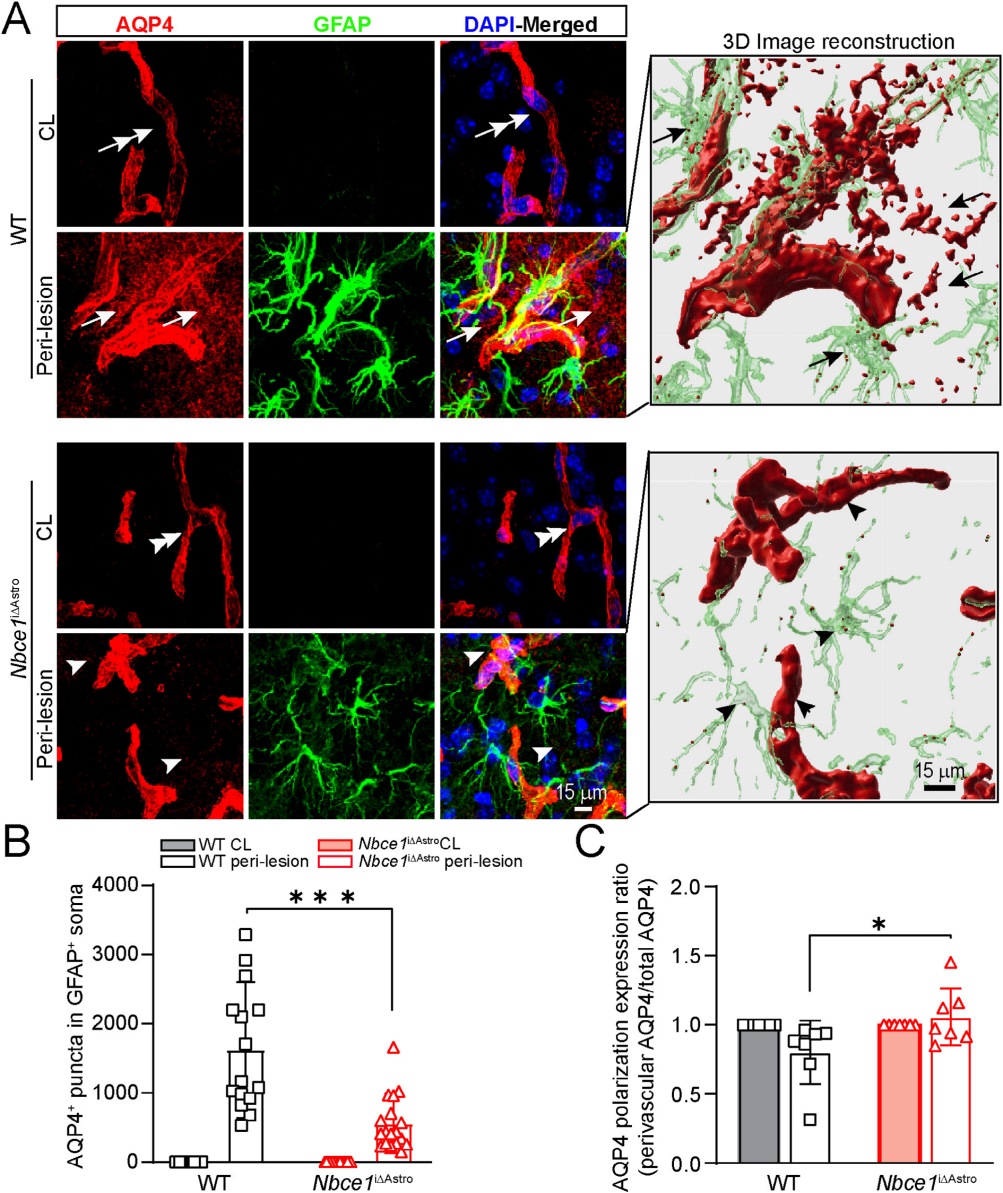
Selective deletion of *Nbce1* in GFAP^+^ astrocytes preserved perivascular AQP4 distribution and reduced its parenchymal expression in stroke brain. (A) Representative confocal images of AQP4 and GFAP immunofluorescence staining in WT and *Nbce1* ^iΔAstro^ brain sections at 3 days post‐stroke. Arrows: High parenchymal AQP4 expression. Arrowheads: Low parenchymal AQP4 expression. Right panel: 3D reconstruction images in A. Arrows: High expression of AQP4 in astrocyte soma and processes. Arrowheads: Low expression of AQP4 in astrocyte soma and processes. (B) Quantification analysis of AQP4^+^ puncta in GFAP^+^ astrocytes. Data are mean ± SD, *n* = 15–18 replicates from *n* = 4 brains; *p < 0.05 compared with WT IL via unpaired *t*‐test. (C) Quantification analysis of perivascular AQP4 polarity. Data are mean ± SD, *n* = 7; *p < 0.05 vs. WT IL via unpaired *t*‐test.

Loss of perivascular AQP4 localization is associated with BBB disruption and cerebral edema (Friedman et al. 2009), and AQP4 water channels are highly concentrated in perivascular astrocyte endfeet as well as in the periventricular and subpial glial limitans and play a significant role in maintaining water equilibrium and BBB integrity (Salman et al. 2022). Focal cerebral ischemia induces cytotoxic edema during the acute phase of stroke, and both pharmacological inhibition and genetic deletion of AQP4 result in improved outcomes and reduced brain swelling in a mouse model of tMCAO (Hirt et al. 2017; Igarashi et al. 2011). To investigate the effect of astrocytic *Nbce1* deletion on perivascular AQP4 expression, we assessed the expression and polarization of AQP4 on astrocytic endfeet in the WT and *Nbce1* ^iΔAstro^ brains at 3 days post‐stroke. As shown in Figure 5A, in the non‐ischemic brain hemispheres, astrocytic AQP4 expression was highly localized to perivascular areas of the cerebral vessels (double arrow) with no detectable GFAP^+^ reactive astrocytes. However, in the WT ischemic peri‐lesion areas, AQP4 protein expression was increased and diffusely distributed in parenchymal regions (white arrows; Figure 5A), accumulating within astrocytic soma and processes (black arrows, 3D reconstruction images). In contrast, the *Nbce1* ^iΔAstro^ stroke brains showed preserved AQP4 localization to cerebral vessels and reduction in parenchymal astrocytes (arrow heads; Figure 5A, 3D reconstruction images). Analysis of the GFAP^+^ cells indicated a significant decrease of AQP4 puncta counts in the reactive astrocytic soma (Figure 5B) but a higher AQP4 polarization expression ratio (perivascular AQP4/total AQP4) in *Nbce1* ^iΔAstro^ stroke brains (Figure 5C). These results clearly suggest that astrocytic NBCe1 activation is linked to dysregulated polarity of AQP4 expression in ischemic brains.

### 3.6 Increased Kir4.1 Protein and Gene (*Kcnj10*) Expression and Reduced Astrocyte Volume Were Detected in *Nbce1* ^iΔAstro^ Stroke Brains

**FIGURE 6:**
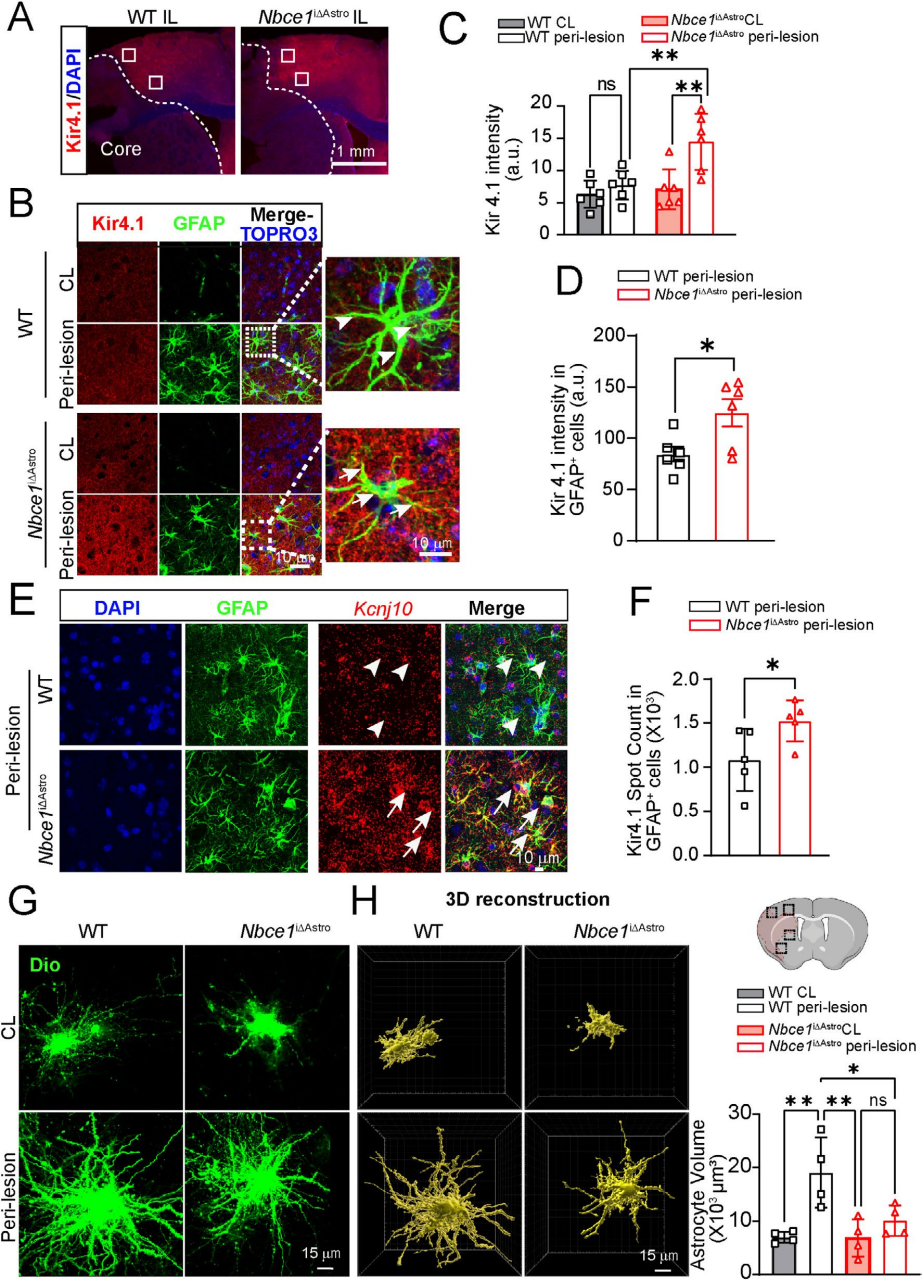
Kir4.1 expression is upregulated in ischemic peri‐lesion areas of *Nbce1* ^iΔAstro^ stroke brains. (A) Representative low magnification immunofluorescence images showing Kir4.1 expression in WT and *Nbce1* ^iΔAstro^ stroke mice. Sampling areas in the ischemic peri‐lesion are shown by the white squares. (B) Representative confocal images of Kir4.1 and GFAP immunofluorescence staining in WT and *Nbce1* ^iΔAstro^ brain sections at 3 days post‐stroke. Arrows: High Kir4.1 expression in astrocytes. Arrowheads: Low Kir4.1 expression. (C, D) Quantification of Kir4.1 signals in CL and IL hemispheres. Data are mean ± SD, *n* = 5. *p < 0.05, **p < 0.01 versus the indicated group via two‐way ANOVA followed by Sidak's multiple comparisons (C) and unpaired *t*‐test (D). (E) Representative confocal images showing *Kcnj10* (Kir4.1) mRNA and GFAP expression in WT and *Nbce1* ^iΔAstro^ mice in the peri‐lesion areas at 3 days post stroke. Arrows: High expression; Arrow heads: Low expression. (F) Quantification of *Kcnj10* transcript spots in GFAP^+^ cells. Data are mean ± SD, *n* = 5. *p < 0.05 via unpaired *t*‐test. G. Representative confocal images of WT and *Nbce1* ^iΔAstro^ stroke brain sections showing Dio labeled astrocytes in the ischemic peri‐lesional areas. (H) 3D reconstruction and quantification of astrocyte volume using IMARIS software. Data are mean ± SD, *n* = 5. *p < 0.05, **p < 0.01 versus the indicated group via two‐way ANOVA followed by Sidak's multiple comparisons.

To understand the mechanisms underlying the reduced BBB permeability in *Nbce1* ^iΔAstro^ stroke brains, we quantified another astrocyte end‐feet specific marker, Kir4.1 (inwardly rectifying potassium channel). Kir4.1 channels are enriched at astrocytic endfeet, where they co‐localize with AQP4, regulate K^+^ spatial buffering, and help limit the osmotic astrocyte swelling (Dibaj et al. 2007; Nagelhus et al. 2004). We examined changes in Kir4.1 mRNA and protein expression in the WT and *Nbce1* ^iΔAstro^ stroke brains. Immunofluorescence analysis revealed that the basal expression of Kir4.1 protein was maintained in the CL hemispheres of both WT and *Nbce1* ^iΔAstro^ stroke brains (Figure 6A–C). Compared to the CL hemispheres, the ischemic peri‐lesion areas in both WT and *Nbce1* ^iΔAstro^ stroke brains exhibited increased Kir4.1 protein expression (Figure 6B,C). Notably, *Nbce1* ^iΔAstro^ brains demonstrated significantly increased Kir4.1 protein expression levels compared to the WT brains (*p* < 0.05, Figure 6A,B). Analysis of Kir 4.1 protein expression in GFAP^+^ cells revealed a significant increase in Kir 4.1^+^ puncta in the soma and processes of the *Nbce1* ^iΔAstro^ brains (*p* < 0.05, Figure 6C,D). Protein expression was corroborated by fluorescence in situ hybridization, which revealed low levels of *Kcnj10* transcripts in GFAP^+^ astrocytes within the peri‐lesional of WT brains (Arrow heads; Figure 6E). However, a significantly increased *Kcnj10* mRNA spot counts were detected in the cell soma and processes of *Nbce1* ^iΔAstro^ stroke brains (Figure 6E,F), implying that NBCe1 deficiency led to upregulating Kir 4.1 gene and protein. Loss of Kir4.1 protein expression has been reported to contribute to glial cell swelling in the post‐ischemic retina (Nwaobi et al. 2016). To assess ischemic astrocyte morphological changes, we analyzed cell volume alterations in GFAP^+^ reactive astrocytes. No change in the expression of the astrocyte reactive marker protein GFAP between the IL hemispheres of WT and *Nbce1* ^iΔAstro^ brains was detected (Figure S10). Analysis of the astrocyte domain organization and soma volume changes in the ex vivo thick stroke brain slices (150 μm) was performed using diolistic labeling, a method previously described for accurately quantifying astrocyte soma volume (Stokum et al. 2018; Waxman et al. 2025). Astrocytes were identified with GFAP immunolabeling, and z‐stacks of diolistic labeled astrocytes were acquired with confocal microscopy. Diolistic labeling revealed fine details of astrocyte morphology, including soma, primary branches, higher‐order branches, and end‐feet. As shown in Figure 6G, cortical astrocytes in WT and *Nbce1* ^iΔAstro^ CL hemispheres exhibited characteristic spongiform morphology with finely branched processes. Quantification of astrocyte volume within individual astrocyte domains showed a mean astrocyte volume in CL hemispheres of WT and *Nbce1* ^iΔAstro^ brains of ~11.7 ± 0.51 × 10^3^ μm^3^, a value comparable to previous reports (Stokum et al. 2018). In the ischemic peri‐lesion areas of WT and *Nbce1* ^iΔAstro^ brains at 3 days post‐stroke, diolistic labeled reactive astrocytes exhibited hypertrophic morphology with increased soma and process volume. In WT stroke brains, diolistic labeled astrocytes displayed swollen soma and processes (Figure 6G), with a mean astrocyte volume of ~27.07 ± 0.75 × 10^3^ μm^3^ (Figure 6H). In contrast, the astrocyte volume in *Nbce1* ^iΔAstro^ stroke brains was significantly reduced (15.0 ± 0.39 × 10^3^ μm^3^) at 3 days post‐stroke (*p* < 0.05; Figure 6G,H). Together, our results indicate that astrocytes lacking NBCe1 show increased expression of Kir4.1 and reduced astrocyte volume, reflecting an adaptive response to preserve homeostatic function under ischemic conditions.

### 3.7 Improved Blood Flow Recovery in *Nbce1* ^iΔAstro^ Stroke Mice During Reperfusion

**FIGURE 7:**
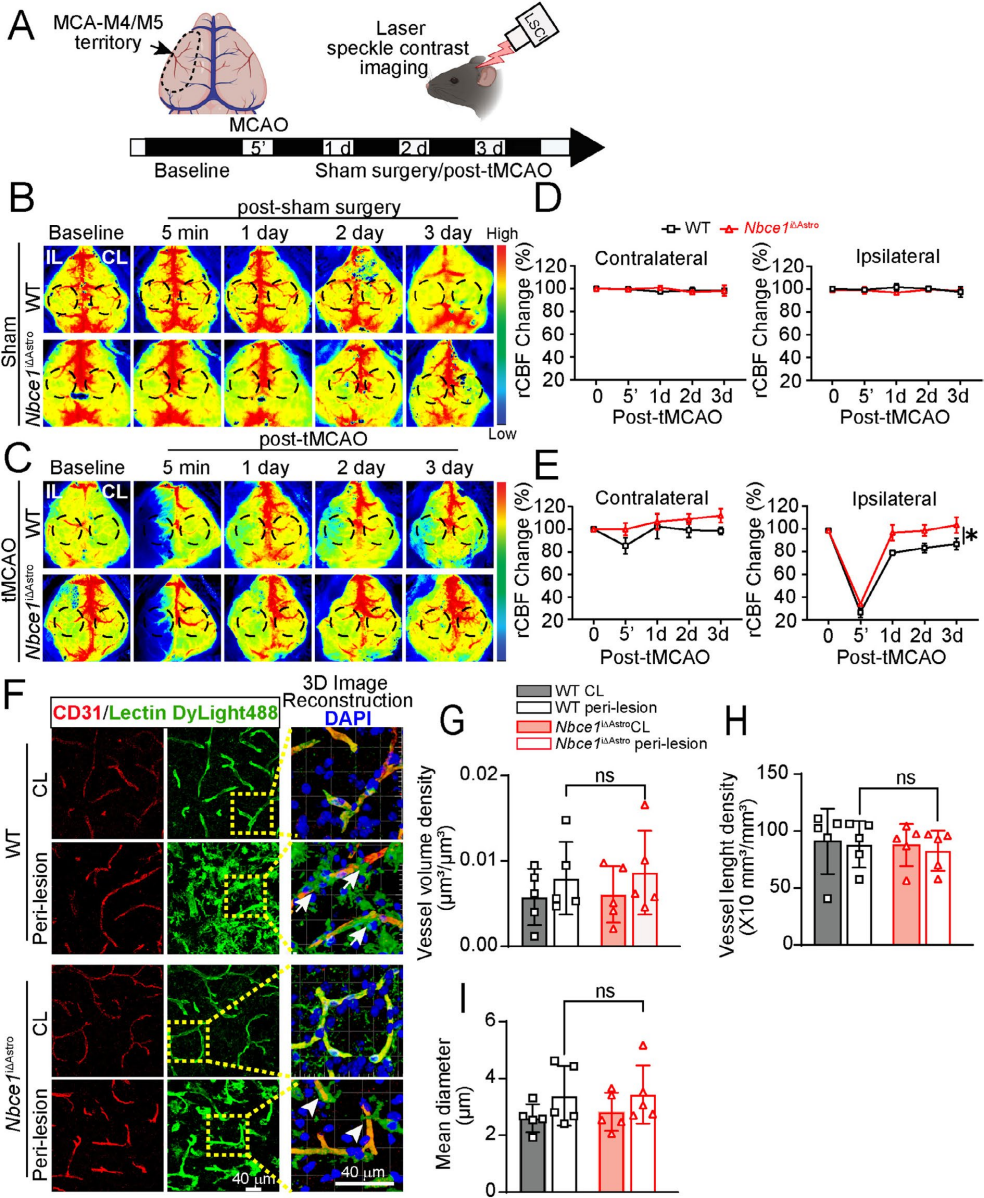
*Nbce1* ^iΔAstro^ mice exhibit faster recovery of regional cerebral blood flow after ischemic stroke. (A) Schematic of experimental design. (B, C) Representative laser speckle imaging analysis of rCBF in the WT and *Nbce1* ^iΔAstro^ mice prior to sham (B) or tMCAO (C) surgeries. Images shown are for baseline, at 5 min, 1‐, 2‐, or 3‐days post‐sham surgery or tMCAO. Dashed circles indicate the regions of interest for quantification of blood perfusion in MCA regions. (D) Summary analysis of rCBF changes as a percentage of pre‐ischemic baseline in CL and IL hemispheres. Data are mean ± SD, *n* = 3. (E) Summary analysis of rCBF changes as a percentage of pre‐ischemic baseline in CL and IL hemispheres. Data are mean ± SEM, *n* = 4; *p < 0.05 vs. WT IL via two‐way ANOVA followed by Sidak's multiple comparisons. (F) Representative confocal images and 3D image reconstructions of CD31 immunostaining and lectin staining in peri‐lesional cortex of WT and *Nbce1* ^iΔAstro^ brains at 3 days post‐stroke. (G–I) Quantification of vessel volume density, length density, and diameter in CL and IL hemispheres of WT and *Nbce1* ^iΔAstro^ stroke brains. Data are mean ± SD, *n* = 5; ns = not significant via two‐way ANOVA followed by Sidak's multiple comparisons. Data are mean ± SEM, *n* = 5; *p < 0.05 vs. WT via unpaired *t*‐test.

Given the pivotal role of perivascular astrocytes in regulating cerebral vessel integrity and flow, we assessed the effect of astrocytic *Nbce1* deletion on recovery of blood flow to the ischemic territory during reperfusion using a PeriCam laser speckle contrast imaging (LSCI) system (Figure 7A). This technique provides a wide‐field perfusion assessment with high spatial and temporal resolution at a depth of 300–700 μm (Davis et al. 2014). The WT and *Nbce1* ^iΔAstro^ mice subjected to sham surgery did not show any significant changes in regional cerebral blood flow (rCBF) in either CL or IL hemispheres (Figure 3B–D). However, both WT and *Nbce1* ^iΔAstro^ stroke mice displayed a significant decrease (~70%) in the rCBF in the IL hemispheres immediately upon tMCAO induction (Figure 7C,E). The WT stroke mice displayed an IL rCBF of 80.4% ± 1.4% of the baseline during 1 day and 88.1% ± 4.9% at 3 days post‐stroke. Compared to the WT mice, the *Nbce1* ^iΔAstro^ mice showed faster rCBF recovery in the IL hemispheres at 1 day (98.1% ± 6.8%) and 3 days post‐stroke (104.7 ± 6.8; *p* < 0.05). However, quantitative 3D imaging analysis of the cerebral vessels immunostained for CD31 and labeled with fluorescent lectin DyLight‐488 revealed no differences in vessel volume density, length density, or diameter changes in ischemic brains of WT and *Nbce1* ^iΔAstro^ mice (Figure 7F–I), indicating the improved cerebral perfusion seen in *Nbce1* ^iΔAstro^ stroke mice is likely not due to gross vascular alterations.

We assessed the extent of inflammation by conducting a protein array analysis of 40 mouse cytokines and chemokines in WT and *Nbce1* ^iΔAstro^ stroke brain homogenates (Figure S11A,B). A modest increase in IL‐1*α*, IL‐*β* and IL1Ra was detected in the IL hemispheres of *Nbce1* ^iΔAstro^ brains compared to the WT IL hemisphere (Figure S11C). Specifically, the increase in TIMP‐1 expression (~1.53 ± 0.67‐fold) in *Nbce1* ^iΔAstro^ IL brains was statistically significant (Figure S11C,D). TIMP‐1 is primarily expressed in astrocytes, and its increased expression during the acute phase of stroke is known to preserve astrocytic endfoot attachment to the endothelium and subsequent repair of tight junctions via *β*1 integrin mediated mechanism (Tang et al. 2020; Tang et al. 2024; Tejima et al. 2009). These results further suggest that the preserved astrocyte function and improved cerebral perfusion seen in *Nbce1* ^iΔAstro^ stroke mice may be mediated through reduced perivascular inflammation.

## 4 Discussion

### 4.1 Conditional Deletion of *Nbce1* Gene in *GfapCre* ^ERT2^ ^+/−^; *Nbce1* ^fl/fl^ Mice Displayed Reduced Ischemic Brain Damage

Recent studies have highlighted the critical role of NBCe1, a major HCO_3_^−^ transporter predominantly expressed in astrocytes, in regulating cell volume, intracellular Na^+^ and HCO_3_^−^ levels, osmotic balance, and pH buffering in the brain (Kurtz 2014). Under physiological conditions, through NBCe1‐mediated transport, astrocytes release bicarbonate to buffer extracellular H^+^ loads associated with increases in neuronal activity (Theparambil et al. 2020). To date, the role of NBCe1 protein in ischemic brain damage remains unclear. Human‐induced pluripotent stem cells (hiPSCs) derived astrocytes showed increased NBCe1 protein expression at 24 h after exposure to in vitro ischemic conditions (Yao et al. 2016). In our study, we detected upregulation of NBCe1 protein in the ischemic peri‐lesion areas of both the cortex and striatum, accompanied by increased neuronal loss and severe neurological functional deficits. Using conditional deletion of the *Nbce1* gene in *GfapCre* ^ERT2+/−^; *Nbce1* ^fl/fl^ mice, we show that *Nbce1* gene deletion occurred in a subset of GFAP^+^ reactive astrocytes, constituting ~30% of all astrocytes. *Nbce1* ^iΔAstro^ stroke mice displayed significant neuroprotective effects, including reduced ischemic stroke volume and hemispheric swelling, reduced BBB damage, increased cerebral blood flow, improved neuronal survival, and enhanced post‐stroke neurological function recovery. These findings suggest that overstimulation of NBCe1 protein function is detrimental in ischemic stroke brains.

A recent study using a mouse model of photothrombotic ischemic stroke demonstrated that selective loss of NBCe1 in astrocytes of *Aldh1l1Cre* ^ERT2+/−^; *Nbce1* ^fl/fl^ mice resulted in increased infarct volume and exacerbated BBB damage, as well as increased production of the pro‐inflammatory CCL2 and toxic NO (Ye et al. 2024). The causes of the discrepancy between our two studies are unclear but may be due to several factors, including the use of two different Cre lines and the two different stroke models. The GFAP‐Cre^ERT2^ mouse line used in our study is widely validated for investigating reactive astrocyte‐mediated regulation of tissue pathology and the underlying molecular mechanisms, particularly in CNS injury (Novakovic et al. 2023; O'Shea et al. 2024; Rorex et al. 2024). This line leverages GFAP upregulation as a hallmark of reactive astrocytes, enabling selective targeting after injury and capturing the full spectrum of astrocyte reactivity. In contrast, the Aldh1l1 Cre line targets homeostatic astrocytes but also affects peripheral immune cells (Amatruda et al. 2025), enteric glia, and Aldh1l1 positive cells in kidney and liver (Li et al. 2023). Ye et al. (2024) reported near complete astrocyte targeting using Aldh1l‐Cre^ERT2^ line; however, this line has also been shown to delete *Nbce1* broadly in the cortex, including neurons (Majumdar et al. 2008), raising concerns about astrocyte specificity and potentially explaining discrepancies between studies.

In our study, we show that deleting the *Nbce1* gene specifically in a subset of GFAP^+^ reactive astrocytes (~30% of all astrocytes) in the ischemic penumbra resulted in significant neuroprotective effects in mice following tMCAO. These findings suggest that our approach of targeting a specific subset of reactive astrocytes in the ischemic penumbra is effective because it is a critical region for stroke progression. However, the photothrombotic stroke model exhibits minimal penumbral region and is unable to dissect the roles of reactive astrocytes in the complex dynamics of injury progression during reperfusion.

### 4.2 Mechanisms Underlying NBCe1‐Mediated Damage

In astrocytes, NBCe1 exhibits bidirectional operation, functioning in both inward and outward modes depending on the concentration gradient of HCO_3_^−^ ions and membrane polarization (Brookes and Turner 1994). Under conditions of membrane depolarization and elevated extracellular HCO_3_^−^, NBCe1 operates in an inward mode, facilitating HCO₃^−^ influx, which reduces intracellular acidification but promotes Na^+^ entry and contributes to astrocyte swelling (Larsen and MacAulay 2017). In mouse neocortex organotypic slice cultures, acute ischemia triggered an inward mode activation of NBCe1, resulting in increased Na^+^ influx and aggravating the ischemia‐induced Na^+^ loading of astrocytes (Everaerts et al. 2023). Besides weakening the driving force of Na^+^‐dependent transporters, NBCe1‐mediated Na^+^ influx also results in a larger decline in cellular ATP levels (Everaerts et al. 2023), which can further aggravate cellular energy failure. In addition, astrocytes can support cell survival and tissue repair by modulating inflammation, thereby influencing the extent of ischemic damage and the efficiency of reperfusion (Liu et al. 2024; Xie and Liu 2023). In our study, cytokine protein array analysis revealed a significant upregulation of TIMP‐1 expression in Nbce*1* ^iΔAstro^ IL hemispheres. TIMP‐1 is a matrix metalloprotease inhibitor shown to be dysregulated in stroke brains and play crucial roles in protecting against BBB disruption (Fujimoto et al. 2008; Tang et al. 2020; Tang et al. 2024). TIMP1 is primarily expressed in astrocytes, and its overexpression by intraperitoneal injection of recombinant mouse TIMP1 led to an increased *β*1 integrin expression in astrocytes, and this, in turn, led to the preservation of astrocytic endfoot attachment to the endothelium and BBB protection following subarachnoid hemorrhage in mice (Tang et al. 2024). In our study, increased TIMP‐1 expression in *Nbce1* ^iΔAstro^ stroke hemispheres may also contribute to the preservation of BBB integrity. However, a trend toward increased expression of IL1*α*, IL1*β* and IL1ra (not statistically significant) was also detected in *Nbce1* ^iΔAstro^ stroke brains. These changes are common in complex inflammatory responses, and are not simple upregulators of pro‐inflammatory mediators. The concurrent elevation of IL1Ra, an endogenous antagonist of IL‐1 signaling, may represent a compensatory mechanism that helps to counterbalance the effects of increased IL1*α* and IL‐1*β*.

### 4.3 AQP4 Polarization in Astrocytic End‐Feet and BBB Damage

AQP4 is a water channel protein predominantly expressed on perivascular astrocytic endfeet. This polarized distribution of AQP4 plays a crucial role in maintaining water homeostasis and BBB integrity (Gao et al. 2020; Iliff et al. 2012). Ischemic stroke induces rapid upregulation and redistribution of AQP4 to parenchymal astrocyte processes, facilitating cell swelling and aggravating BBB damage (Zador et al. 2009). In a mouse model of tMCAO, AQP4 expression was rapidly upregulated in perivascular endfeet as well as in parenchyma, reaching a peak at 1 h post occlusion, coinciding with early cerebral swelling in the core and border of the lesion (Ribeiro Mde et al. 2006). A recent study by Stokum et al. found that ischemia triggers the calmodulin‐dependent translocation of AQP4 to the plasma membrane following SUR1‐TRPM4 and NCX1 activation in astrocytic endfeet (Stokum et al. 2023). Pharmacological inhibition or astrocyte‐specific deletion of SUR1‐TRPM4 or NCX1 reduced cytosolic AQP4 expression, brain swelling, and improved neurological function in mice (Stokum et al. 2023). This underscores the coordinated role of ion channels, transporters, and water channels in astrocytes during stroke (Stokum et al. 2018; Stokum et al. 2023). In our study, *Nbce1* ^iΔAstro^ stroke brains exhibited preserved AQP4 localization to the cerebral vessels, with reduced expression in parenchymal astrocytes. In contrast, in the WT stroke brains, AQP4 localization was more disrupted, with increased localization in the cell soma, suggesting that astrocytic NBCe1 plays a role in the dysregulation of AQP4 expression polarity in the ischemic brain. Interactions between AQP4 channels and ion transporter proteins result in hetero‐multimeric structures that can regulate the expression, cell membrane localization, and gating of the AQP4 protein (Connors and Kofuji 2006). While such interactions have been documented for several endfeet proteins, the potential formation of complexes between NBCe1 and AQP4 and their impact on perivascular localization is unknown and needs further investigation. Our results highlight the complex interplay between NBCe1 and AQP4 in astrocytes during ischemic conditions and suggest that targeting NBCe1 could be a potential strategy for preserving AQP4 polarity and mitigating stroke‐induced brain edema.

### 4.4 The Selective Deletion of *Nbce1* in Reactive Astrocytes Also Triggers Compensatory Mechanisms

The compromised K^+^ homeostasis in the ischemic penumbra disrupts the electrochemical gradients necessary for efficient glutamate uptake by astrocytes, further contributing to excitotoxicity and neuronal injury (Leis et al. 2005; Milton and Smith 2018; Nwaobi et al. 2016). In this regard, homeostatic feedback mechanisms are crucial for restoring balance and mitigating damage. Kir4.1 channels in astrocytes are crucial for spatial K^+^ buffering, a clearance mechanism for excessive extracellular K^+^ in tripartite synapses and facilitation of glutamate uptake via coupling to glutamate transporters (Ohno et al. 2021). Reduced expression or impaired activation of Kir4.1 channels has been implicated in acute brain injuries and various neurological disorders (Bataveljic et al. 2024; Hong et al. 2023; Ohno et al. 2021; Tong et al. 2014). In a tMCAO mouse stroke model, a significant reduction in Kir4.1 channel currents was detected in NG2 glia in the hippocampus, resulting in impaired K^+^ uptake and clearance (Hong et al. 2023). Upregulation of NBCe1 is reported to contribute to Kir4.1 channel dysfunction, resulting in impaired K^+^ uptake and motor neuron hyperexcitability (Barbay et al. 2024). In our study, we detected an increased expression of Kir4.1 protein in ischemic peri‐lesion areas of *Nbce1* ^iΔAstro^ stroke brains, associated with a significant reduction in astrocyte domain volume. Our results suggest that upregulation of Kir4.1 channels in *Nbce1* ^iΔAstro^ stroke brains could be a compensatory response to help maintain K^+^ homeostasis and regulate astrocyte volume. Enhanced Kir4.1 expression may improve astrocytic K^+^ buffering and osmotic regulation, along with glutamate clearance from the peri‐lesion, thereby limiting damage. This could in part underlie the reduced neuronal loss in *Nbce1* ^iΔAstro^ stroke brains. Further studies are needed to define how NBCe1 regulates Kir4.1 expression and whether this impacts K^+^ buffering.

Beyond neuronal protection, astrocyte‐mediated support is also critical for maintaining cerebral vascular integrity. The potential contribution of enhanced collateral perfusion in *Nbce1* ^iΔAstro^ stroke mice cannot be ruled out. Investigation into CBF changes in WT and *Nbce1* ^iΔAstro^ stroke brains through autoregulatory mechanisms and their impact on stroke outcomes warrants further study.

## 5 Conclusions

Our novel findings show that astrocytic NBCe1 protein plays a critical role in brain injury in the tMCAO model of ischemic stroke. Using inducible, astrocyte‐specific *GfapCre‐ER* ^+/−^; *Nbce1* ^fl/fl^ conditional knockout mice, we demonstrated that selective deletion of *Nbce1* in ^+^GFAP‐reactive astrocytes reduced infarct volume, brain swelling, and accelerated recovery of neurological function. This was accompanied by reduced vascular inflammation, preserved perivascular AQP4 polarization, and reduced BBB permeability. These results suggest that the NBCe1 protein in astrocytes is a key mediator of cellular and tissue damage during ischemic stroke, potentially through its effects on pH regulation, ion homeostasis, and cellular swelling. Our findings identify astrocytic NBCe1 as a promising target for regulating perivascular astrocyte dysfunction and improving BBB integrity in stroke brains.

## Author Contributions

O.C., G.B., and S.M.T. designed the study. O.C., S.K., and G.B. analyzed data and wrote the manuscript. G.B., O.C., S.K., K.K., M.A., M.MF, E.B., R.M., and S.F. carried out most experiments. S.M. helped with IMARIS image analysis. S.S. performed MCAO surgeries for the MRI study. L.M.F. and T.K.H. performed the MRI. V.F. helped with transgenic mouse breeding, colony maintenance, genotyping, and manuscript editing. S.W. and I.A.S. helped with DiOlistic Labeling. All authors discussed the results and commented on the manuscript.

## Conflicts of Interest

The authors declare no conflicts of interest.

## Supporting information


**Data S1:** Supporting Information.

## Data Availability

The data that support the findings of this study are available from the corresponding author upon reasonable request.
