# Supplementary material for: Selective Deletion of NBCe1 in Reactive Astrocytes Attenuates Ischemic Stroke Brain Damage
Source: Glia. 2025 Aug 5;73(12):2386–406. doi: 10.1002/glia.70075 (PMC12541896; doi:10.1002/glia.70075)
Supplement: Supplementary file 1 — Data S1: Supporting Information. [file GLIA-73-2386-s001.docx]

**Supplementary Information**

**Selective Deletion of NBCe1 in Reactive Astrocytes Attenuates Ischemic Stroke Brain Damage**

Okan Capuk^1^, Elise Berthold^1^, Kathiravan Kaliyappan^1^, Mansi Avunoori^1^, Rajesh Muduganti^1^, Sanjana Krishna^2^, Shamseldin Metwally^1^, Mary McFarland^1^, Shanshan Song^1, 7^, Victoria Fiesler^1, 7^, Sydney Fischer^1^, Lesley M Foley^3^, T Kevin Hitchens^3, 4^, Susannah Waxman^5^, Ian A Sigal^5^, Shefeeq M Theparambil^6^, Gulnaz Begum^1, 7*^

**Supplementary Figures**


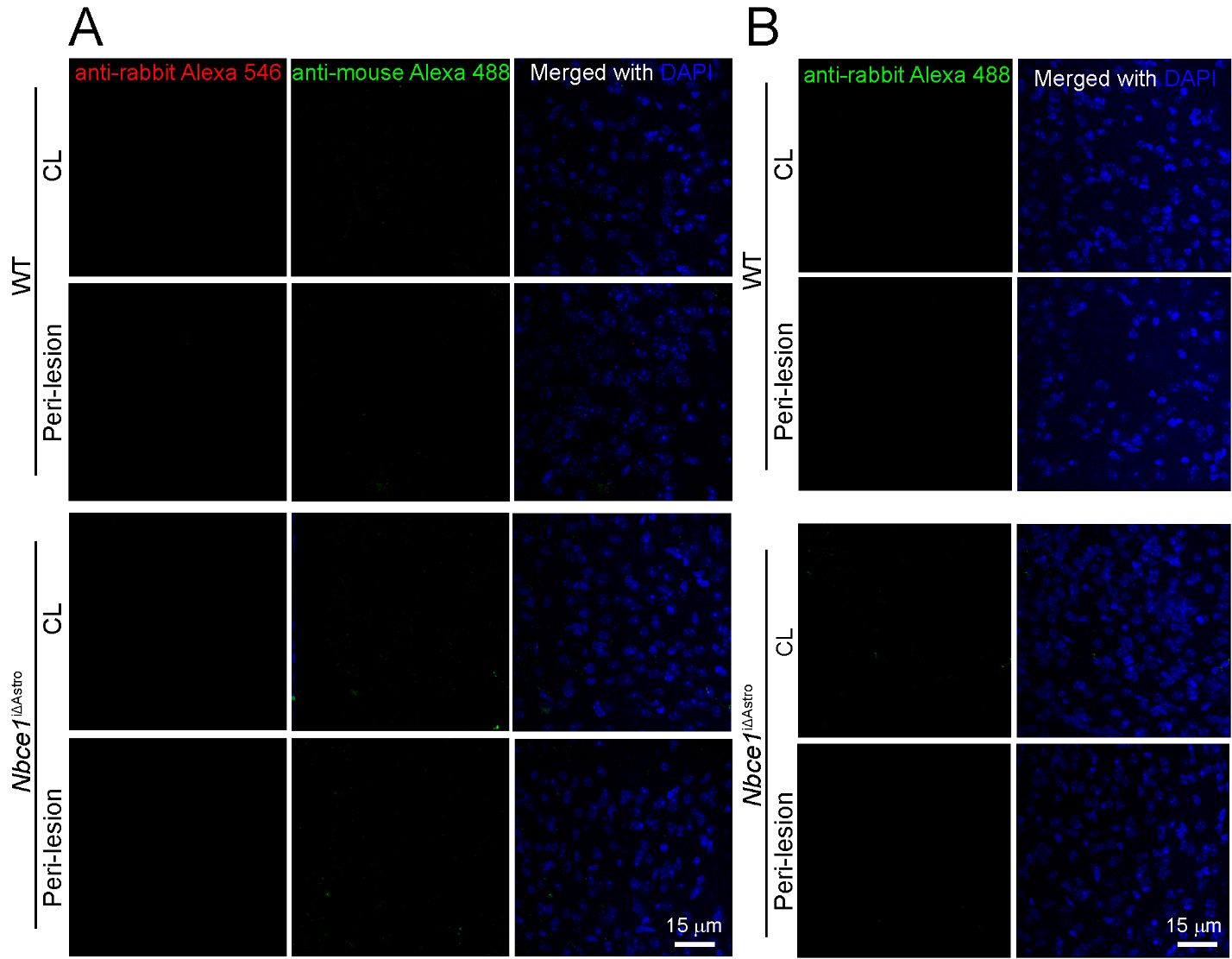


**Figure S1.** A and B. Representative secondary antibody only (Goat anti-rabbit Alexa 546 and anti-mouse 488; Goat anti-rabbit Alexa 488) treated negative control images from WT and *Nbce1*^iΔAstro^ stroke brain sections at 3 days post-stroke.


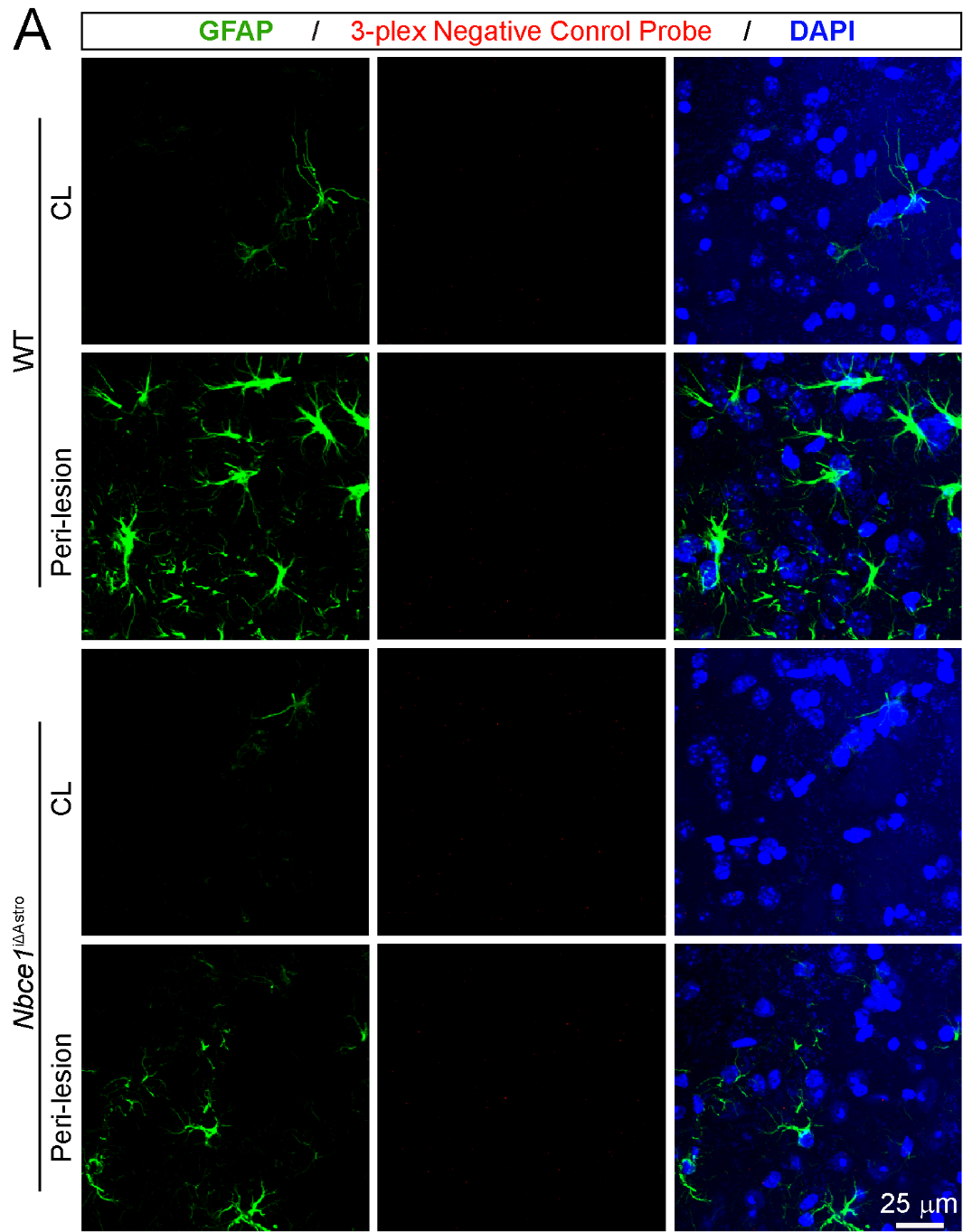


**Figure S2.** A. Representative RNAscope 3-plex negative control probe treated images with GFAP immunostaining from WT and *Nbce1*^iΔAstro^ stroke brain sections at 3 days post-stroke.


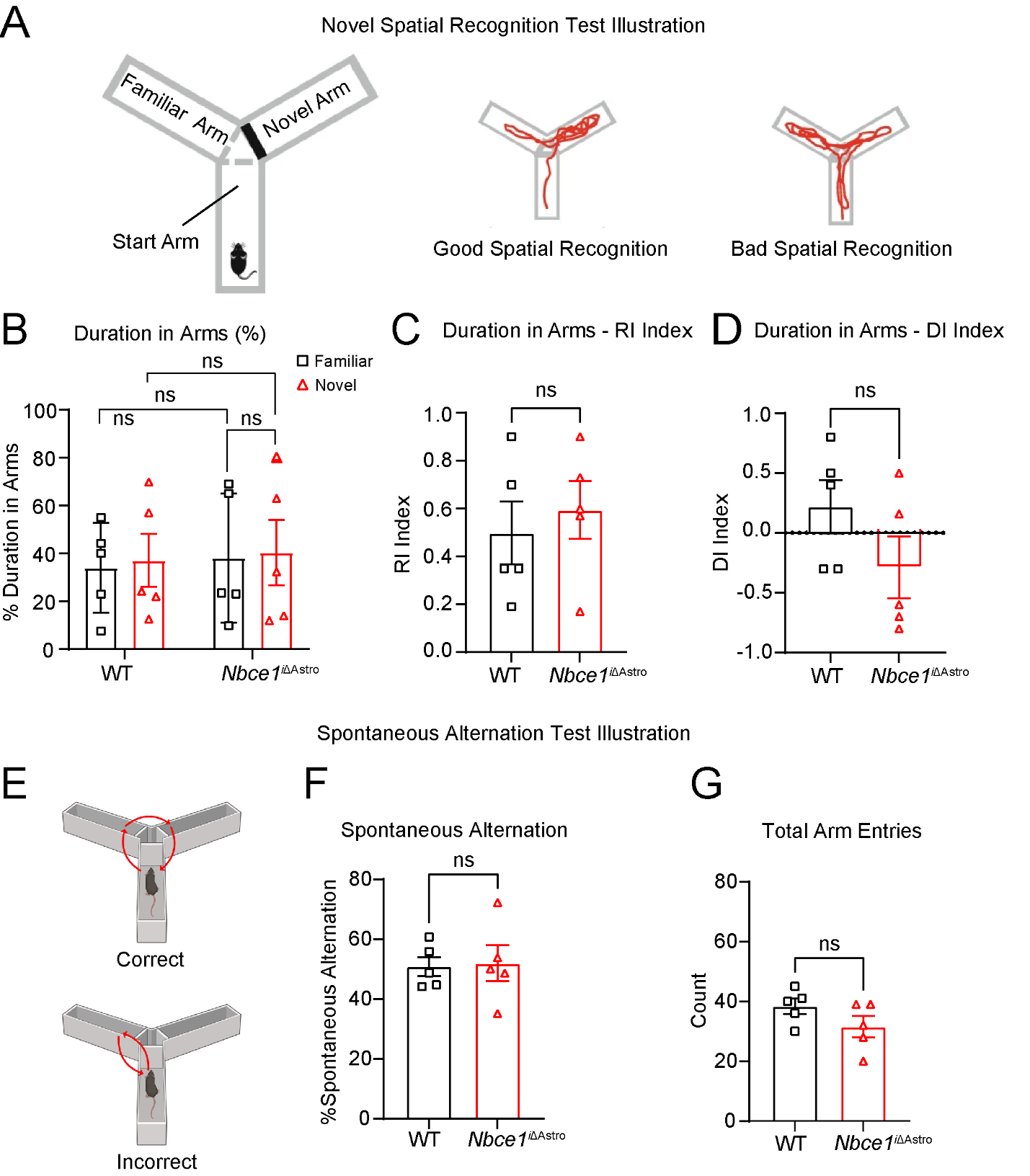


**Figure S3. Y-maze novel object recognition test and spontaneous alternation test** **between WT and *Nbce1*^iΔAstro^ naïve mice.** A. Illustration of Y-maze novel object recognition test. B and C. Y-maze novel object spatial recognition test in naïve mice, shows no difference in duration in arms (%), recognition index, and discrimination index. Data are mean +/- SEM. n=5; ns=not significant vs WT via two-way ANOVA, followed by Fisher’s LSD multiple comparisons (B), and unpaired t test (C and D). E. Illustration of Y-maze spontaneous alternation test. F and G. Y-maze spontaneous alternation test in naïve mice, shows no difference in spontaneous alternation (%) and total entries. Data are mean +/- SEM. n=5; ns=not significant vs WT via unpaired t test.


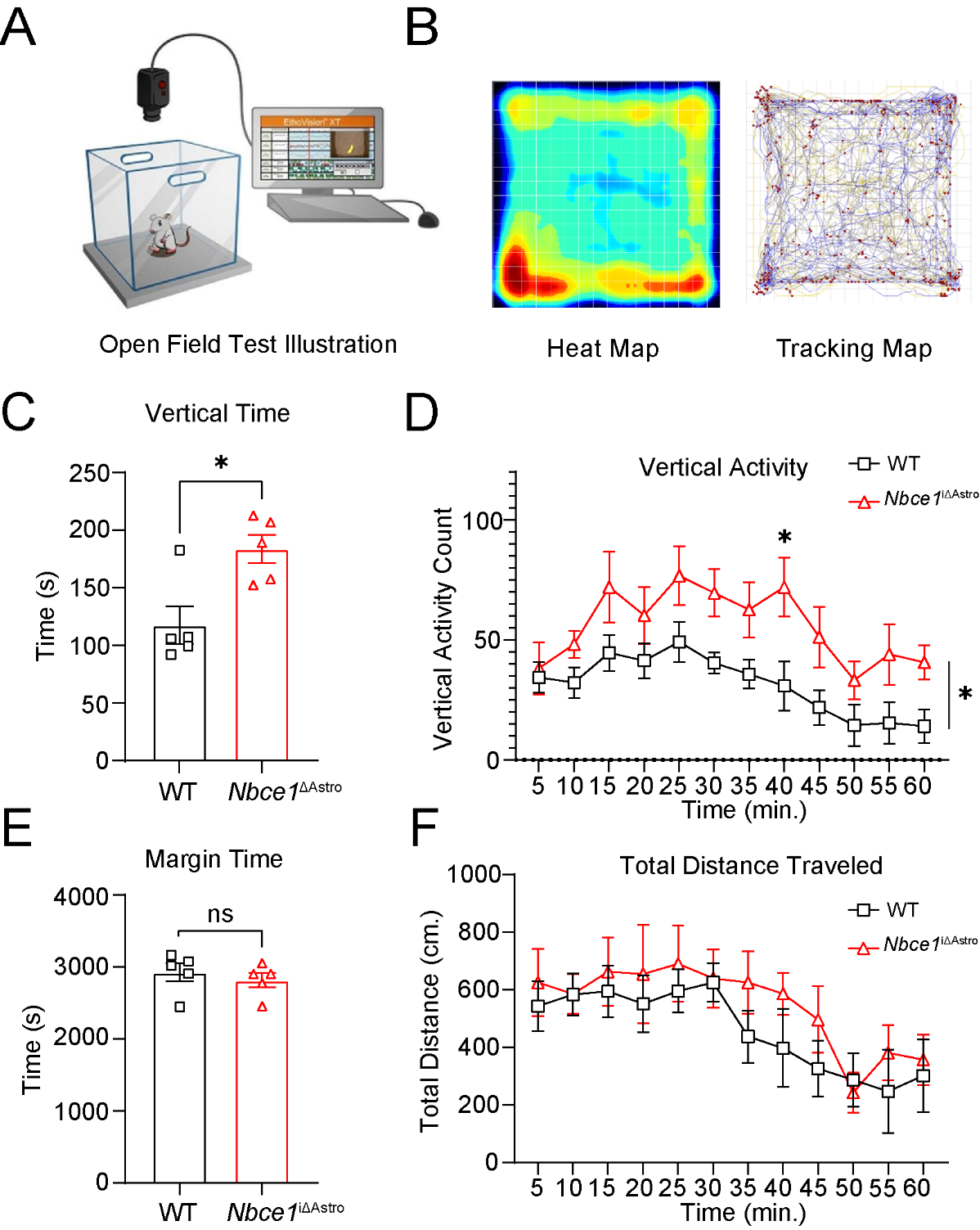


**Figure S4. *Nbce1*^iΔAstro^ naïve mice exhibited increased exploratory behavior in the open field test compared to WT mice.** A-B. Open field test illustration and tracking map examples. C and D. Open field test in naïve mice, shows significant difference in vertical time and vertical activity. Data are mean +/- SEM. n=5; *p<0.05 vs WT via unpaired t test (C) and two-way ANOVA, followed by Sidak’s multiple comparisons (D). E and F. Open field test in naïve mice, shows no difference in margin time and total distance traveled. Data are mean +/- SEM. n=5; ns=not significant vs WT via unpaired t test (E) and two-way ANOVA, followed by Sidak’s multiple comparisons (F).


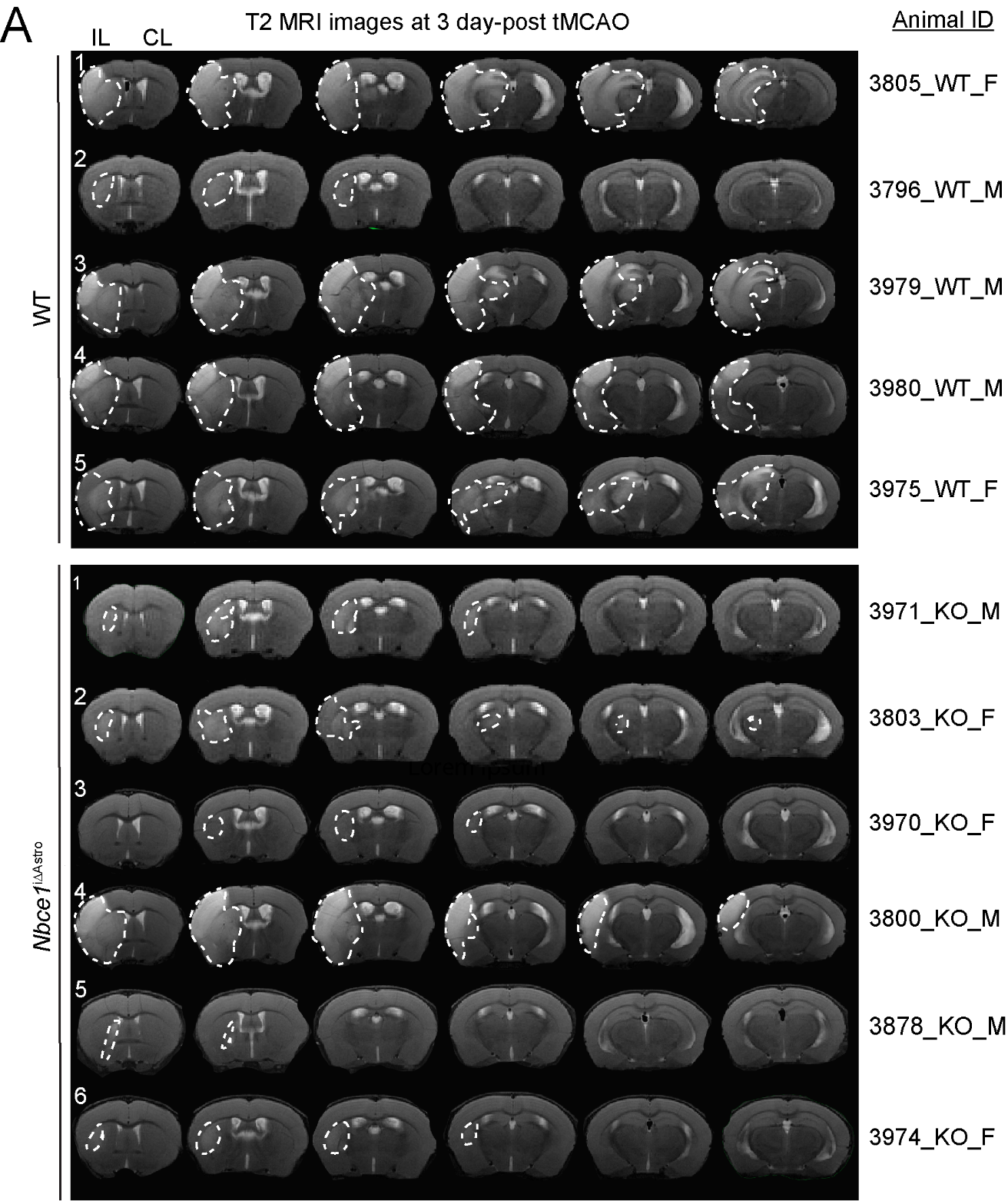


**Figure S5.** T2-weighted MRI brain images of all the WT and *Nbce1*^iΔAstro^ brains 3 days post stroke used for analysis.


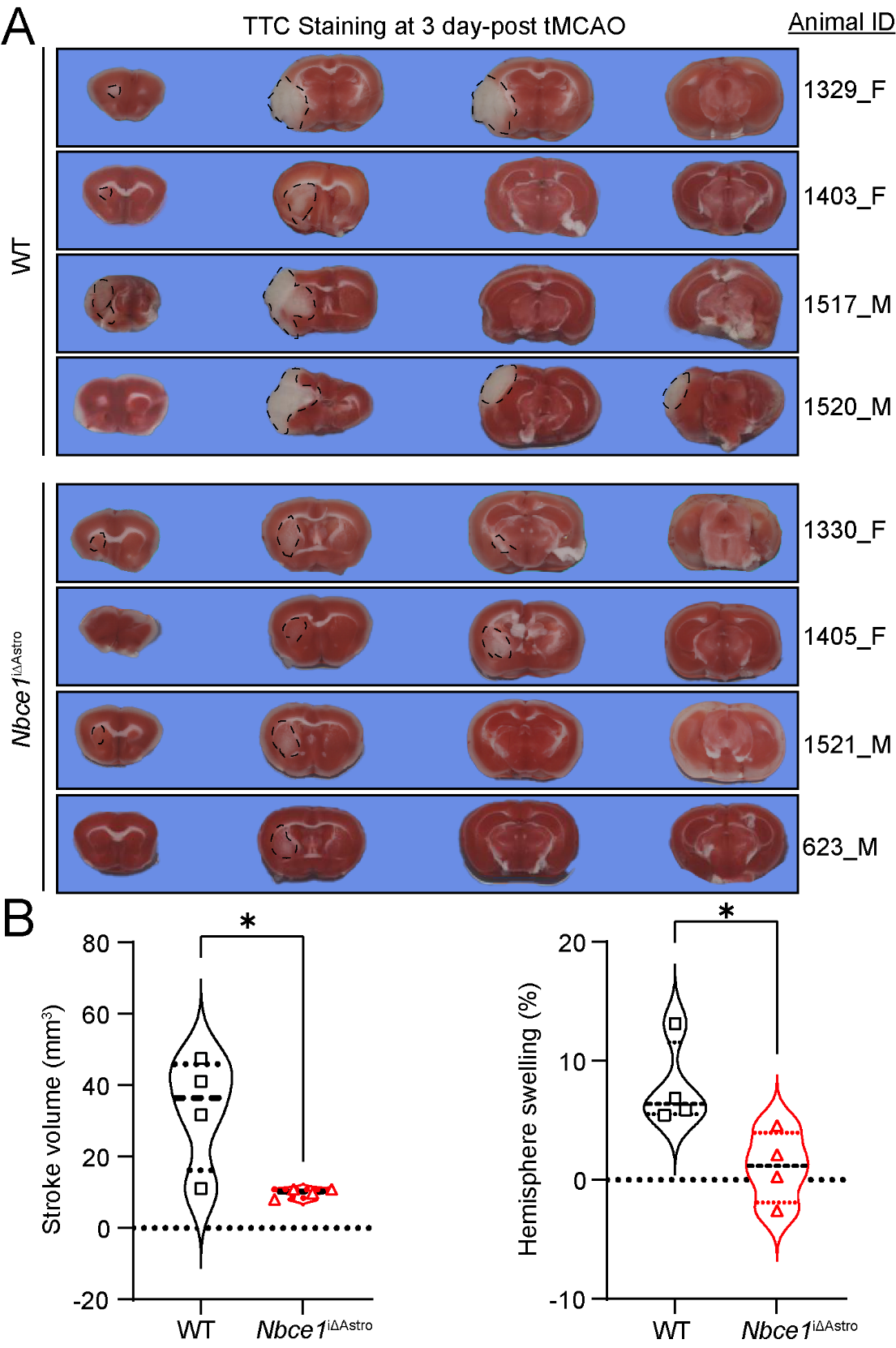


**Figure S6. *Nbce1*^iΔAstro^ mice displayed reduced stroke volume and swelling at 3 days post-stroke.** A. WT and *Nbce1*^iΔAstro^ brain sections stained with TTC (2,3,5-triphenyltetrazolium chloride) at 3 days post-stroke. B. Quantitation of stroke volume and hemispheric swelling (%). Data presented as violin plots. n=4; *p<0.05 vs WT via unpaired t test.


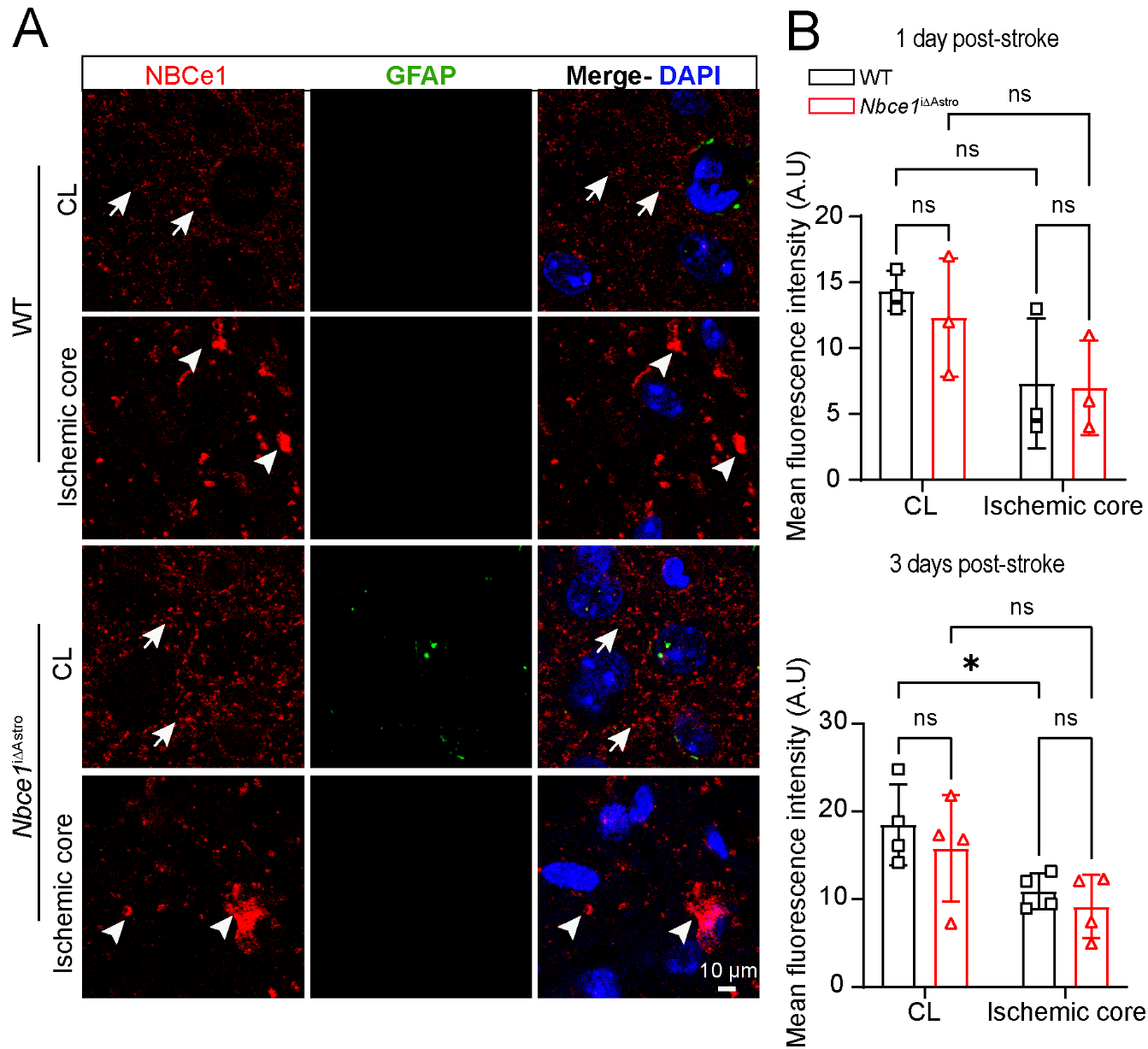


**Figure S7. A.** Representative confocal images showing NBCe1 protein expression in the CL hemisphere and ischemic core of WT and *Nbce1*^iΔAstro^ brains at 1-day post stroke. **B.** Quantification of NBCe1 immunosignal intensity. Data are mean +/- SD, n=3, versus the indicated group via two-way ANOVA followed by Sidak’s multiple comparisons.


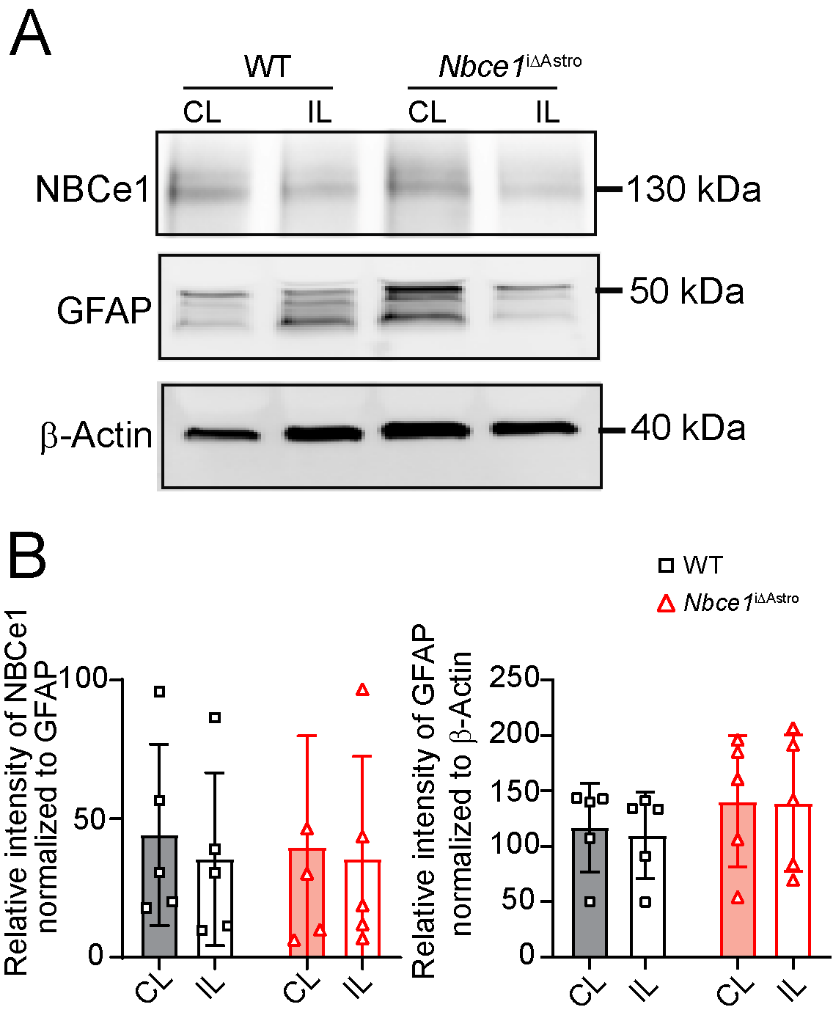


**Figure S8. A.** Representative western blotting image of NBCe1 protein expression in astrocytes isolated from CL and IL hemispheres of WT and *Nbce1*^iΔAstro^ brains at 3-day post stroke. **B**. Quantification of NBCe1/(GFAP/β-actin) and GFAP (GFAP/β-actin) immunosignals. Data are mean +/- SD, via unpaired t-test, n=4.


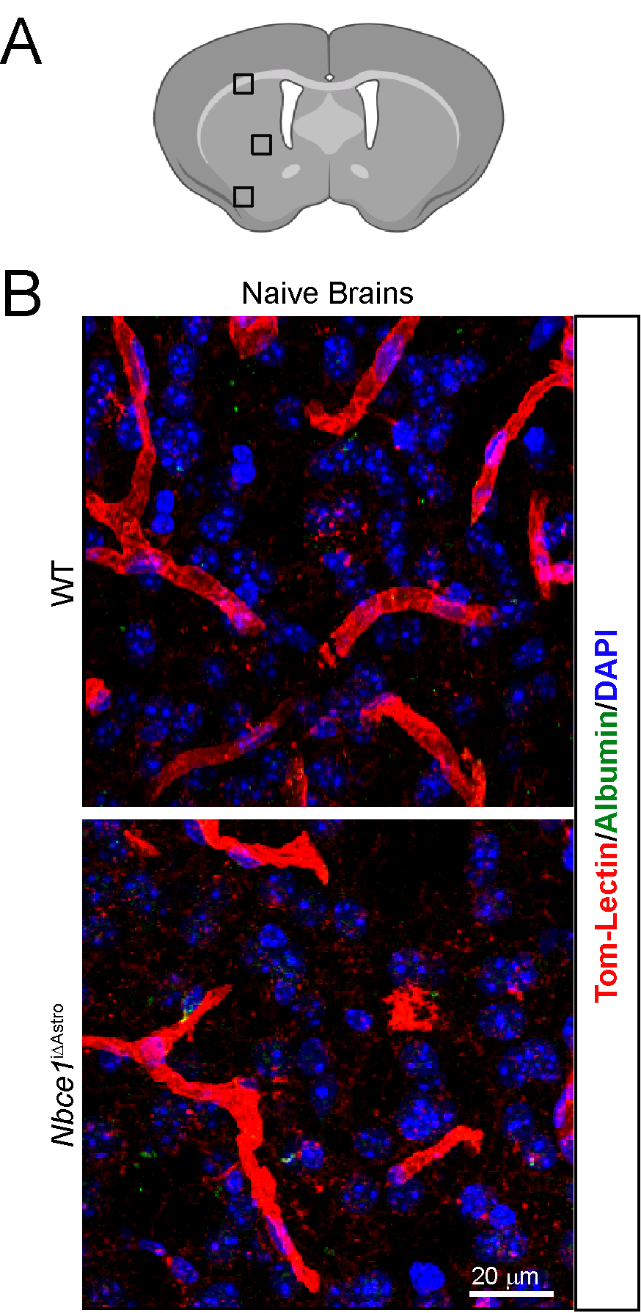


**Figure S9. WT and *Nbce1*^iΔAstro^ naive brains did not show any BBB leakage**. **A.** Schematic of brain images showing the sampling areas. **B.** Representative confocal images of WT and *Nbce1*^iΔAstro^ naive brain sections stained with vessel marker, tomato lectin and Alexa 488 conjugated anti-albumin antibody.


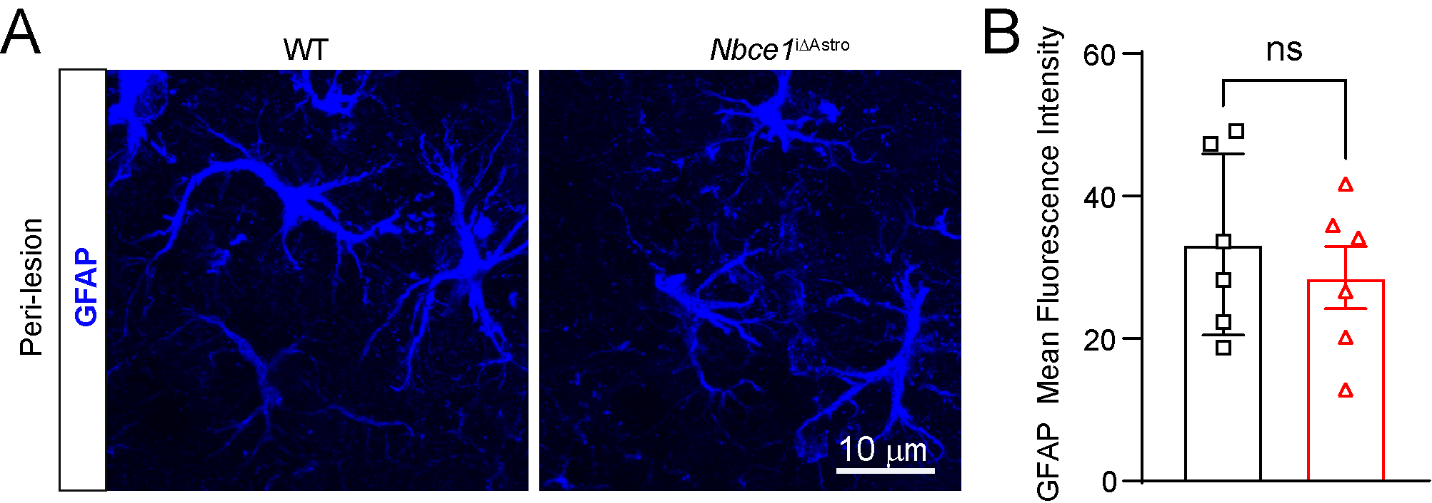


**Figure S10. A.** Representative confocal images of WT and *Nbce1*^iΔAstro^ brains sections showing GFAP protein expression in the peri-lesion areas at 3-day post stroke. **B**. Quantification of GFAP signal intensity. Data are mean +/- SD, via unpaired t-test, n=6.


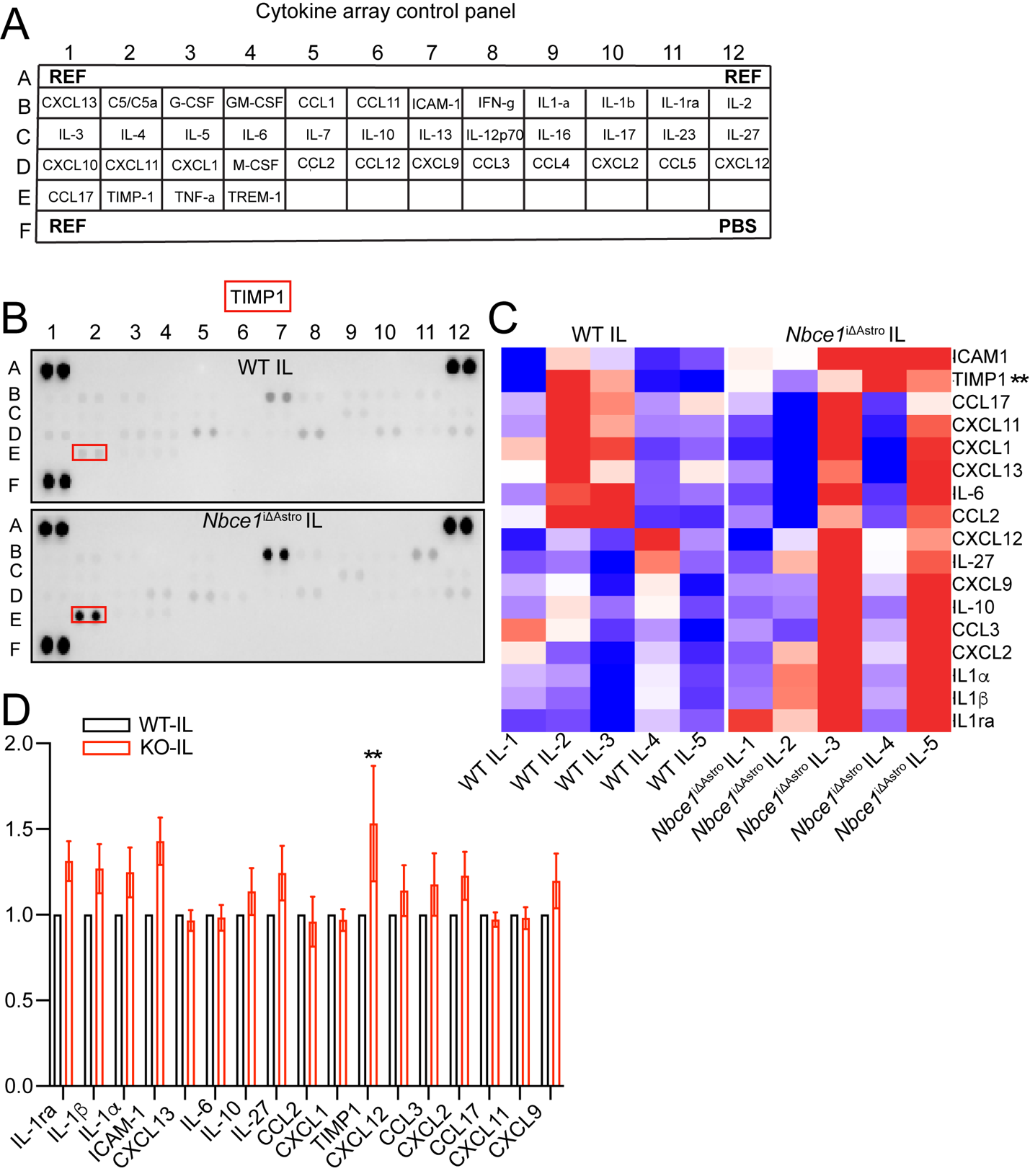


**Figure S11. *Nbce1*^iΔAstro^ mice displayed reduced inflammatory cytokine and chemokine expression at 3 days post-stroke. A.** Reference key for cytokine array, adapted from the manufacturer’s information. **B.** Representative array images probed with protein homogenate samples from WT and *Nbce1*^iΔAstro^ IL hemispheres. **C.** Heatmap of cytokine and chemokine expression from IL hemispheres of WT and *Nbce1*^iΔAstro^ stroke brains. **D.** Summary of fold change expression in *Nbce1*^iΔAstro^ IL hemispheres normalized to WT IL hemispheres. Data are mean ± SEM. n=5; **p<0.01 vs WT via two-way ANOVA followed by Sidak’s multiple comparisons

**Supplemental Table 1. List of antibodies used for immunofluorescence staining (IF), Western blotting (WB) and flow** **cytometry.**

| **Antibody** | **Host** | **Dilution** | **Company** | **Catalog/RRID Number** | **Application** |
| --- | --- | --- | --- | --- | --- |
| anti-SLC4A4 | Rabbit | 1:200 | Proteintech | #11885/AB_2191458 | IF |
| anti-GFAP | Mouse | 1:200 | Cell signaling | #3670/AB_561049 |  |
| anti-GFAP | Rabbit | 1:200 | Agilent | #Z0334/AB_10013382 |  |
| Anti-GFAP  Anti-AQP4  Anti-Neun  Anti-Kir 4.1  CD31 (PECAM-1)  Alexa anti rabbit 488  Alexa anti rabbit 546  Alexa anti mouse 488  Alexa anti chicken 488  Tomato Lectin 488  DAPI  TO-PRO^TM^-3 Iodine | Chicken  Rabbit  Rabbit  Rabbit  Rabbit  Goat  Goat  Goat  Chicken  -  -  - | 1:200  1:200  1:100  1:200  1:100  1:200  1:200  1:200  1:200  2,5:200  1:1000  1:1000 | Abcam  EMD Millipore  Abcam  Alomone Labs  Cell Signaling  Invitrogen  Invitrogen  Invitrogen  Invitrogen  Vector  Sigma-Aldrich  Invitrogen | #ab4674/AB_304558  #AB3594/AB_91530  #ab177487/AB_2532109  #APC-035/AB_2040120  #77699/AB_2722705  #A11008/AB_143165  #A11035/AB_2534093  #A11029/AB_2534088  #A48260/AB_2890271  #DL-1174/AB_2336404  #D1306/No RRID number  #T3605/No RRID Number |  |
| anti-SLC4A4  anti-GFAP  anti-rabbit HRP | Rabbit Rabbit  Goat | 1:300  1:1000  1:5000 | Proteintech Agilent  Bio-Rad | #11885/AB_2191458  #Z0334/AB_10013382  #1706515/AB_11125142 | WB |
| PE anti-mouse CD8a | Rat | 1:266 | BioLegend | 100707/AB_312746 | Flow Cytometry |
| Anti-mouse ACSA-2 | Human | 1:266 | Miltenyi Biotec | # 130-116-243/AB_ 2727421 |  |
|  |  |  |  |  |  |

**Supplemental Table 2.** Parameters for T2WI

| MRI machine | Repetition Time (ms) | Effective Echo Time (ms) | Matrix | Field of View | RARE factor | Slice thickness  (mm) | No of slices |
| --- | --- | --- | --- | --- | --- | --- | --- |
| Bruker | 4000 | 40 | 256x256 | 20x20 for a 0.078 x 0.078 mm in-plane resolution | 8 | 0.5 | 25 |
